# Retinal biomarkers in cognitive impairment and dementia: Structural, functional, and molecular insights

**DOI:** 10.1002/alz.70672

**Published:** 2025-09-10

**Authors:** Yan Min, Hongyu Zhou, Zixiao Li, Yongjun Wang

**Affiliations:** ^1^ Department of Neurology Beijing TianTan Hospital Capital Medical University Beijing China; ^2^ China National Clinical Research Center for Neurological Diseases Beijing China; ^3^ Research Unit of Artificial Intelligence in Cerebrovascular Disease Chinese Academy of Medical Sciences Beijing China

**Keywords:** cognitive impairment, dementia, imaging techniques, retinal biomarkers

## Abstract

Cognitive impairment and dementia, including Alzheimer's disease (AD), pose a global health crisis, necessitating non‐invasive biomarkers for early detection. This review highlights the retina, an accessible extension of the central nervous system (CNS), as a window to cerebral pathology through structural, functional, and molecular alterations. By synthesizing interdisciplinary evidence, we identify retinal biomarkers as promising tools for early diagnosis and risk stratification. Nevertheless, translating biomarkers into clinical practice faces significant translational hurdles, including methodological heterogeneity, confounding variables, lack of standardized protocols, and insufficient longitudinal validation in diverse populations. Overcoming these challenges through rigorous standardization and robust validation studies is essential to establish the clinical utility of retinal biomarkers.

**Highlights:**

- Stage‐specific diagnostic potential: Retinal biomarkers (e.g., OCT/OCTA changes, eye movements, molecular deposits) show alterations correlating with cognitive impairment stages (preclinical AD to dementia), assisting stage differentiation.
- Reflecting cerebral pathology: Multimodal retinal alterations (structural, functional, molecular) correlate with key brain pathologies (amyloid/tau burden, atrophy, small vessel disease), indicating shared pathways.
- Translation gaps and future: Clinical adoption faces barriers (method heterogeneity, confounders, limited validation). Future requires rigorous screening of candidate retinal biomarkers, extensive multicenter validation, and artificial intelligence (AI)‐facilitated clinical translation.

## 1 INTRODUCTION

Cognitive impairment and dementia, particularly Alzheimer's disease (AD), represent a major global health challenge, primarily affecting aging populations. Currently, over 55 million individuals worldwide live with dementia, a figure projected to triple by 2050.^1^ In the United States alone, approximately 6.9 million older adults have AD, with healthcare costs exceeding $360 billion annually. Beyond economic impact, the burden includes significant caregiver strain; 11 million unpaid caregivers provided 18.4 billion hours of care in 2023, and reduced quality of life for patients and families.^2^ Characterized by amyloid‐beta (Aβ) plaques, tau neurofibrillary tangles, and neurovascular dysfunction, AD remains incurable, with irreversible neuronal damage often occurring before clinical diagnosis.^3^ Early detection during preclinical stages is considered to offer an opportunity to alter disease trajectories, with evidence suggesting that delaying onset by one year could reduce global burden by 9 million.^4^

Conventional diagnostic tools, including cerebrospinal fluid (CSF) biomarkers, amyloid positron emission tomography (PET), and magnetic resonance imaging (MRI), face limitations related to invasiveness, high cost, and the need for specialized infrastructure, making them less practical for widespread screening.^5^, ^6^, ^7^ The retina, an embryological extension of the central nervous system (CNS) sharing developmental origins, microvascular architecture, and barrier systems with the brain, provides a potential approach for observing cerebral pathology.^8^, ^9^, ^10^ Retinal ganglion cells (RGCs) and microvasculature mirror neurodegenerative changes observed in AD,^11^ including Aβ deposition, neuroinflammation, and synaptic loss,^12^, ^13^ with associated axonal degeneration in the optic nerves.^14^

Advances in retinal imaging have enabled precise quantification of structural, functional, and molecular biomarkers.^15^ Optical coherence tomography (OCT) can detect retinal and choroidal thickness (CT), correlating with structural brain imaging alterations,^16^ while OCT angiography (OCTA) may detects early‐stage microvascular deficits in AD.^17^ Functional assessment such as dynamic vessel analysis (DVA) identifies impaired neurovascular coupling, and molecular assays can identify Aβ and inflammatory biomarkers in ocular fluids.^18^, ^19^ Other noninvasive techniques, including electroretinography (ERG) to assess RGC dysfunction,^20^ retinal oximetry for altered oxygen metabolism,^21^ visual evoked potentials (VEPs) for cortical processing deficits,^22^ and eye movement tracking to detect executive dysfunction,^23^ have suggested correlations with cognitive impairment. These methods provide complementary insights into functional and pathological changes associated with cognitive impairment. However, study variability in methodologies, equipment, and participant demographics highlights the need for standardized protocols.^12^ This review consolidates current evidence on retinal biomarkers across structural, functional, and molecular modalities in cognitive impairment. It evaluates associations between multimodal retinal imaging and clinical stages of cognitive impairment alongside neuroimaging pathology, analyzes current limitations and challenges, and proposes potential strategies to facilitate clinical translation.

## 2 RETINAL BIOMARKERS

This section synthesizes findings on retinal parameters, including ophthalmic assessments, fundus diseases, and multimodal imaging techniques for structural, functional, and molecular evaluation across clinical stages and subtypes of cognitive impairment. All retinal biomarkers discussed are summarized in Table 1.

**TABLE 1:** Cognitive‐related retinal biomarkers.

Structural biomarkers		
Routine ophthalmic parameters		Intraocular pressure^24^; pupil size; VA^25^; contrast sensitivity^26^; stereopsis^27^; visual field; vision‐specific functioning, and vision‐specific mobility^28^
Fundus diseases		Cataract^29^
		AMD^30^
		Glaucoma^31^
		DR^32^
		RVO^33^
CFP	Quantitative parameters	CRAE^34^
		CRVE^34^
		AVR^34^
		FD^35^
		Vessel tortuosity^34^
		Vascular network complexity^34^, ^35^
	Qualitative parameters	Microaneurysms; retinal hemorrhages; cotton wool spots and else retinopathy signs^10^
OCT		Retinal layer thickness: RNFL; IPL; INL; IFT; ONL; RPE; GC‐IPL; GCC; GCL^16^, ^17^, ^36^, ^37^
		CT^37^, ^38^
		Macular volume^16^, ^37^, ^38^
OCTA		VD; CD; PD; VLD; VPD; VSD^15^, ^17^, ^37^
		FAZ; FR^17^, ^37^, ^39^
Functional biomarkers		
DVA		Arterial/venous dilation^40^, ^41^, ^42^
		Arterial/venous dilation amplitude^40^, ^42^
		Reaction time^40^, ^42^
ERG		Amplitude and implicit times of the ERG wave components^15^, ^20^
		PhNR^15^, ^20^
Retinal oximetry		Retinal vessel oxygen saturation^21^
VEPs		Amplitudes of VEP components ^22^
		Latency of VEP components^22^
Eye movements		Saccades (prosaccade; antisaccade; memory saccade)^43^
		Fixation metrics^44^
		Smooth pursuit^45^
Molecular biomarkers		
Curcumin‐based retinal Aβ imaging^46^		
HSI of retinal Aβ^47^		
Detection of Aβ in the lens,^48^ aqueous humor,^49^ and tears^50^		

Abbreviations: Aβ, amyloid‐beta; AMD, age‐related macular degeneration; AVR, arteriole‐to‐venule ratio; CD, capillary density; CFP, color fundus photography; CRAE, central retinal arteriole equivalent; CRVE, central retinal venule equivalent; CT, choroidal thickness; DR, diabetic retinopathy; DVA, dynamic vessel analysis; ERG, electroretinography; FAZ, foveal avascular zone; FD, fractal dimension; FR, roundness of the FAZ; GCC, ganglion cell complex; GCL, ganglion cell layer; GC‐IPL, ganglion cell‐Inner plexiform layer; HSI, hyperspectral imaging; IFT, inner foveal thickness; INL, inner nuclear layer; IPL, inner plexiform layer; OCT, optical coherence tomography; OCTA, optical coherence tomography angiography; ONL, outer nuclear layer; PD, perfusion density; PhNR, photopic negative response; RNFL, retinal nerve fiber layer; RPE, retinal pigment epithelium; RVO, retinal vein occlusion; VA, visual acuity; VEPs, visual evoked potentials; VD, retinal vessel density; VLD, vessel length density; VPD, vascular perfusion density; VSD, vessel skeleton density.

### 2.1 Routine ophthalmic parameters and cognition

#### 2.1.1 Visual acuity impairment

Numerous cross‐sectional and longitudinal studies have consistently suggested significant associations between visual impairment (VI) and cognitive impairment. In nationally representative samples, both distance vision deficits and self‐reported VI have been linked to lower cognitive scores,^51^ while moderate‐to‐severe VI increases dementia risk by 1.5‐ to 2‐fold across diverse populations.^25^, ^52^ Meta‐analyses corroborated these findings, showing that VI is associated with a 2.4‐fold increase in dementia odds in cross‐sectional analyses and a 2.1‐fold increase in longitudinal studies.^53^ Longitudinal studies also suggest a bidirectional relationship: baseline VI predicts a 2.3‐fold higher dementia incidence (hazard ratio [HR] = 2.3, 95% confidence interval; [CI]: 2.0–2.6), whereas baseline dementia doubled the likelihood of subsequent VI development.^54^ Notably, individuals with excellent baseline vision exhibited a 63% lower dementia risk over 8.5 years,^55^ while population attributable fractions estimated that 4.9%–19.0% of dementia cases may stem from VI.^56^ Declines in visual acuity (VA) have been shown to parallel declines in Mini‐Mental State Examination (MMSE) scores, with VA deterioration correlating with MMSE decline (*r* = −0.139, *p* = 0.03).^57^ Aligned with these observations, a study of AD patients found that those with mild cognitive impairment (MCI) reported visual difficulties, while those with severe impairment showed moderate VA deficits.^58^ Evidence links VI to lower cognitive scores and increased dementia risk, with bidirectional relationships observed, though potential shared risk factors and the need for mediation analyses remain to be clarified.

#### 2.1.2 Other ophthalmic parameters

In addition to VA, several other visual parameters may offer unique prognostic value. Contrast sensitivity (CS), in particular, has shown stronger longitudinal associations with dementia risk than VA. Worsening CS over time significantly increases dementia risk,^26^ with dementia prevalence being higher among individuals with CS impairment (25.9%) compared to the general sample (12.3%). Adjusted analyses indicate a 31% higher dementia prevalence in individuals with CS deficits compared to unimpaired counterparts.^59^ Additional visual parameters, such as visual field deficits, functional visual limitations, and impaired visual mobility, have also been associated with cognitive decline, dementia, and AD.^28^, ^60^, ^61^, ^62^ AD patients often show reduced stereopsis^27^ and lower intraocular pressure.^24^ Together, these findings suggest that comprehensive ophthalmic assessments, including measures beyond standard VA testing, point to potential directions for further research, particularly regarding CS, which requires dialectical analysis considering fundus and systemic conditions.

### 2.2 Fundus diseases and cognition

#### 2.2.1 Cataract

Cataract, a leading cause of VI in older adults, has been consistently associated with risks of cognitive decline and dementia across longitudinal cohorts and meta‐analyses. Studies with follow‐up periods ranging from 4.1 to 8.4 years report elevated risks of AD and all‐cause dementia among cataract patients, with hazard ratios ranging from 1.28 to 2.41.^63^, ^64^, ^65^ The risk appears to be amplified in individuals with comorbidities such as diabetes or cardiovascular disease, with combined exposures increasing dementia risk by 1.19–2.29 times.^66^ Meta‐analyses support these associations, indicating pooled odds ratios (ORs) of 1.32 for cognitive impairment and relative risks of 1.17–1.23 for all‐cause dementia.^67^, ^68^

The relationship between cataracts and dementia extends to both AD and vascular dementia (VaD), although effect sizes vary. Meta‐analyses report a consistent association with AD (HR = 1.28, 95% CI: 1.13–1.45),^68^ and a significant but more variable association with VaD (HR = 1.35, 95% CI: 1.06–1.73; OR = 1.26, 95% CI: 1.09–1.45).^68^, ^69^ Cataract severity may play a role, with more advanced cases associated with greater odds of cognitive decline (adjusted OR = 1.34) and progressive deterioration in cognitive test scores.^29^, ^70^

The underlying mechanisms linking cataracts to cognitive decline remain unclear, proposed pathways include visual deprivation, shared pathological processes, or common systemic risk factors.^64^, ^65^ Notably, observational studies suggest that cataract surgery may reduce dementia risk by 25%–29%,^71^, ^72^ underscoring the clinical significance of timely intervention in older adults. However, limitations in the available evidence include heterogeneity in findings (especially for VD where effect sizes vary), reliance on observational data susceptible to confounding, and insufficient representation of diverse populations. Future studies should further examine associations for specific dementia subtypes in broader and more diverse populations.

#### 2.2.2 Age‐related macular degeneration

Age‐related macular degeneration (AMD), a common eye condition in older adults, has been widely studied for its association with cognitive function through longitudinal cohort studies and meta‐analyses, though findings should be interpreted with caution. Population‐based cohort studies show modest but consistent associations between AMD and incident dementia. One study reported a 50% increased AD risk among individuals with AMD (HR = 1.50, *p* < 0.05) over 31,142 person‐years of follow‐up,^73^ a finding corroborated in Asian populations (HR = 1.48).^74^ Another cohort study reported a 1.26‐fold increased risk of dementia after adjusting for confounding variables.^66^ Meta‐analyses consolidated these trends, indicating a 27% higher AD risk (RR = 1.27, 95% CI: 1.06–1.52) and a 29% increase in all‐cause dementia risk (RR = 1.29, 1.13‐1.48) associated with AMD.^67^ Cognitive test performance is also poorer among AMD patients across multiple neuropsychological measures.^75^ However, large‐scale studies   have reported no significant associations.^30^ These discrepant results may stem from methodological variations in diagnostic criteria and differences in population characteristics.

Biological plausibility for the AMD‐dementia link is supported by shared pathophysiological mechanisms. Both conditions involve β‐amyloid deposition (in drusen and senile plaques), chronic inflammation, and oxidative stress.^76^ Bidirectional epidemiological relationships have been observed: AMD progression may predict cognitive decline, while dementia may accelerate AMD pathogenesis through neurovascular dysregulation.^77^, ^78^ This reciprocal interaction reflects systemic aging processes rather than simple unidirectional causality.^75^ Therefore, further validation is essential given limitations including insufficient research coverage across cognitive impairment stages and dementia subtypes, and reliance on self‐reported AMD diagnoses that introduce misclassification bias due to > 60% of early AMD cases remaining undetected by patients.^79^

#### 2.2.3 Glaucoma

Evidence from multiple Asian cohort studies suggests an association between glaucoma and increased dementia risk, particularly AD. Studies report a 1.46‐ to 1.5‐fold increased risk of AD within 5 years of glaucoma diagnosis.^31^, ^73^ One cohort found that baseline glaucoma was associated with higher incident dementia (HR = 2.38, 95% CI: 1.08–5.23) and AD (HR = 2.77, 95% CI: 1.17–6.56) after adjustment for confounders.^80^ Meta‐analyses support these findings, showing increased risk of all‐cause dementia (RR = 1.16) and AD (RR = 1.18), with stronger associations observed in women, elderly populations, and patients with primary open‐angle glaucoma.^67^, ^81^ Neuroimaging revealed that glaucoma patients exhibit disrupted functional connectivity in working memory networks alongside visual pathway alterations,^82^ suggesting potential shared neuropathological alterations. Proposed mechanisms may involve dysregulation of CSF pressure^24^ and altered Aβ metabolism.^83^

However, some studies from Europe and North America report inconsistent or null findings.^63^, ^66^ For instance, a large study of 2,431,150 California Medicare beneficiaries found that all open‐angle glaucoma subtypes were associated with lower odds of any dementia compared to no glaucoma.^84^ Such discrepancies may reflect population heterogeneity and methodological differences. Key limitations of existing studies include the preponderance of cross‐sectional designs, which limit causal inference, and insufficient attention on glaucoma subtypes. Further research should involve prospective glaucoma phenotyping and efforts to clarify whether glaucoma‐associated parameters can enhance dementia risk stratification beyond conventional diagnostic approaches.

#### 2.2.4 Diabetic retinopathy

Multiple longitudinal studies suggested a significant association between diabetic retinopathy (DR) and an increased risk of cognitive impairment and dementia. Cohort studies with follow‐up periods ranging from 4 to 12.6 years have reported that individuals with DR are 2.5–3.4 times more likely to experience cognitive decline.^85^, ^86^, ^87^ Notably, DR severity exhibits a dose‐dependent relationship with cognitive impairment, with moderate‐to‐severe DR linked to threefold higher odds of incident cognitive decline.^86^ The population‐based Edinburgh Type 2 Diabetes Study found that increasing DR severity was associated with worse performance on cognitive tests, with moderate‐to‐severe retinopathy linked to the poorest general cognitive ability and individual test results.^88^ While some studies report null associations after adjusting for diabetes status,^63^ pooled estimates from systematic reviews support DR as an independent risk factor, particularly for VaD (OR = 1.68) and AD (adjusted HR = 1.34).^89^, ^90^ These discrepancies may arise from inconsistent DR diagnosis recording, residual confounding due to factors like health care access variation, or varied adjustment for confounders across studies. Structural neuroimaging studies support this association, showing that greater DR severity correlates with reduced gray matter volume and accelerated cognitive decline.^32^

Additional evidence from systematic reviews and meta‐analyses adds to the evidence for this association. Wu et al. reported a pooled OR of 2.45 (95% CI: 1.76–3.41) for cognitive dysfunction in individuals with DR, indicating a statistically association through meta‐analytic synthesis.^91^ Similarly, a review encompassing 2,094 patients found a significantly higher risk of cognitive impairment in DR patients compared to controls.^92^ Cheng and colleagues analyzed data from 15 systematic reviews and 10 meta‐analyses and confirmed a significant positive association between DR and cognitive impairment.^93^ Chan et al. integrated findings from 17 cross‐sectional and 8 longitudinal studies, indicating that DR is linked to both higher cross‐sectional prevalence of cognitive impairment and an elevated longitudinal risk of cognitive decline in diabetic patients.^94^ Collectively, these studies provide consistent evidence linking DR to adverse cognitive outcomes.

Interestingly, cognitive impairment also increases DR risk (OR = 1.76),^89^ suggesting overlapping pathophysiological pathways beyond a simple cause‐and‐effect relationship. The underlying mechanisms may involve neurovascular dysfunction, as both DR and cognitive decline share features of microvascular degeneration, chronic inflammation, glial activation, and oxidative stress induced by hyperglycemia and advanced glycation end‐products.^95^ Although DR has received comparatively more research attention than other ocular diseases regarding its association with cognitive outcomes, current studies still suffer from two key limitations: they fail to incorporate cardiovascular risk factors to examine their potential mediating effects on the DR‐cognition relationship, and there exists methodological heterogeneity in DR staging without dedicated investigation of early‐stage cognitive impairment. Future research requires more longitudinal cohorts that include diverse age groups and varying stages of cognitive impairment. These studies should adjust for multiple confounding factors, such as diseases (both ocular and systemic) and social factors, while establishing clear diagnostic criteria for DR to better interpret its relationship with cognitive function.

#### 2.2.5 Retinal vein occlusion

Retinal vein occlusion (RVO), the second most common retinal vascular disorder,^96^ has been investigated for associations with cognitive impairment and dementia, though epidemiological evidence remains inconsistent. A large US cross‐sectional study found higher dementia prevalence in those with retinal vascular occlusion, but the association became non‐significant after adjusting for age or stroke, suggesting shared vascular risk factors rather than an independent link.^33^ In contrast, a large Korean nationwide cohort study showed a significant longitudinal association: RVO patients had increased risks of all‐cause dementia (HR = 1.16), AD (HR = 1.15), and VaD (HR = 1.24) after adjusting for confounders including hypertension.^97^ Adding to evidence for an independent link, another cross‐sectional study found RVO associated with higher odds of all‐cause dementia and VaD (OR = 3.29), with this association seen in both central and branch RVO subtypes.^98^ However, a large Danish prospective cohort indicated age‐dependent risk: RVO was linked to a 9% higher dementia risk in those under 75, but an 8% lower risk in those over 75 at exposure.^99^ Inconsistencies exist regarding the independence of the RVO‐dementia association from confounders, the association with AD, and the impact of age.

Future research requires prospective longitudinal studies featuring detailed phenotyping of RVO and dementia, rigorous adjustment for systemic and genetic risk factors and inclusion of diverse populations. Determining whether RVO functions as an independent biomarker for dementia risk stratification constitutes a critical objective for clinical research.

### 2.3 Parameters from fundus structural examination

Alterations in retinal structural biomarkers derived from color fundus photography (CFP), OCT, and OCTA reflect underlying neuropathology associated with cognitive impairment, and show potential for differentiating dementia subtypes (Table 2).

**TABLE 2:** Key studies on the association between retinal structural biomarkers and cognitive function.

Author	Cohort information	Sample	Retinal parameter	Age	Gender character (Female, %)	Retinal imaging	Cognitive outcome	Summary of results
CFP parameters correlated with cognitive function								
Gatto et al.,^100^	Observational case–control study *n* = 809 USA	All: 809	Retinopathy; Retinal vascular caliber	All: 70.7 (7)	All: 59%	Topcon TRC 50EX Retinal Camera	Subjects with generalized arteriolar narrowing had twice the odds of a low CASI‐S score	Retinal calibers and any retinopathy were not associated with the cognitive factors as continuous variables
Ding et al.,^101^	Observational case–control study *n* = 954 UK	All: 954	Retinal vascular caliber	All: 67.2 (4)	All: 49%	Non‐stereoscopic color photographs	Enlarged retinal arteriolar/venular were significantly associated with lower scores for the Wechsler Logical Memory test	Enlarged retinal arteriolar/venular calibers linked to impaired memory, especially in men
Baker et al.,^102^	Observational case–control study *n* = 2211 USA	All: 2211 (dementia: 159)	Retinopathy; Retinal vascular caliber	All: 74 (4)	All: 59%	Fundus camera	Persons with retinopathy had lower mean DSST scores	Retinopathy and focal arteriolar narrowing were associated with dementia risk in hypertensive individuals
Kim et al.,^103^	Observational case–control study *n* = 2452 USA	All: 2452	Retinopathy; Retinal vascular caliber	All: 79.5 (4)	All: 60%	Fundus camera	NA	High micro‐/macrovascular burden individuals had the poorest cognitive function
de Jong et al.,^104^	Longitudinal cohort study *n* = 5553 Netherlands	11.6 years follow up: 655 dementia (AD: 519 and vascular dementia: 73)	Retinal vascular caliber	All: 67.7 (8)	All: 59%	Fundus camera (Topcon Optical Company)	NA	Larger venular calibers were tied to vascular dementia; smaller arteriolar calibers were linked to increased risk of vascular dementia only when adjusted for venular calibers
Schrijvers et al.,^105^	Observational case–control study *n* = 195 Netherlands	11.4 years follow up: AD^f^: 149 VaD: 29 Other subtypes of dementia: 17	Retinopathy	All: 81.6 (8)	All: 69%	Topcon TRV‐50VT fundus camera	NA	Retinopathy linked to prevalent dementia (OR = 2.04), similar in AD/vascular types; no incident dementia association during 11.4‐year follow‐up
Cheung et al.,^106^	Observational case–control study *n* = 195 Singapore	AD^b^: 136 HC: 290	Retinal vascular caliber; Fractal dimension; Tortuosity	AD: 74.8 (6) HC: 73.9 (5)	AD: 53% HC: 47%	Fundus camera	NA	Narrower venular caliber, lower arteriolar/venular fractal dimension, and higher tortuosity linked to AD
Jinnouchi et al.,^107^	Observational case–control study *n* = 1053 Japan	Dementia: 351 HC: 702	Retinopathy; Retinal vascular caliber	Dementia: 67.9 (0.4) HC: 67.8 (0.3)	Dementia: 64% HC: 64%	Fundus camera	NA	Generalized arteriolar narrowing was associated with an increased risk of disabling dementia
Wong et al.,^108^	Observational case–control study *n* = 8733 USA	Retinopathy Absent: 8143 Retinopathy Present: 590	Retinopathy	Retinopathy Absent: 57.3 Retinopathy Present: 54.5	Retinopathy Absent: 52% Retinopathy Present: 51%	Fundus camera	Retinopathy was associated with Delayed Word Recall Test, Digit Symbol Subtest, and Word Fluency Test	Cognitive function scores ≤‐2SD linked to higher microaneurysms, hemorrhage, and soft exudates
Lesage et al.,^109^	Longitudinal cohort study *n* = 803 USA	All: 803	Retinopathy	All: 58.4	All: 60%	Fundus camera	Those with retinopathy had WF decline per decade (vs. no decline without) and more rapid DSS decline	Individuals with retinopathy showed declines in executive function
Deal et al.,^110^	Longitudinal cohort study *n* = 12,313 USA	All: 12,317	Retinopathy	All: 60 (6)	All: 44%	Fundus camera	NA	Loss of retinal vascular integrity was associated with greater 20‐year decline
Deal et al.,^111^	Longitudinal cohort study *n* = 12,482 USA	All: 12,482	Retinopathy; Retinal vascular caliber	All: 60.5 (6)	All: 56%	Nonmydriatic fundus cameras	NA	Moderate/severe retinopathy and narrow central retinal arterioles linked to all‐cause dementia
Lee et al.,^112^	Observational case–control study *n* = 2624 USA	All: 2624	Retinopathy; Retinal vascular caliber	All: 76.1 (5)	All: 58%	Fundus camera	NA	Retinal hemorrhages doubled all‐cause MCI/dementia odds
El Husseini et al.,^113^	Longitudinal cohort study *n* = 4334 USA	4334 participants	Retinal vascular caliber	All: 61.6 (9)	All: 53%	Digital nonmydriatic camera	Retinal vascular calibers were associated with cognitive function	Larger CRAE and CRVE linked to lower cognitive scores
O'Neill et al.,^114^	Observational case–control study *n* = 2624 Ireland	MCI^c^: 156 HC: 1275	Retinal vascular caliber	MCI: 64.4 (9) HC: 62.1 (8)	MCI: 45% HC: 53%	Canon CX‐1 Digital Fundus Camera	NA	No significant associations between retinal microvascular parameters and MCI were detected
Cheung et al.,^115^	Longitudinal cohort study *n* = 491 Singapore	Cognitive decline: 254 HC: 237	Retinal vascular caliber	Cognitive decline: 73.8 (7) HC: 70.7 (8)	Cognitive decline: 48% HC: 42%	Canon CR‐DGi 10D or Canon CR‐1 40D	NA	Deep‐learning‐based measurement of retinal vessel caliber was associated with risk of cognitive decline and dementia
Frost et al.,^116^	Observational case–control study *n* = 148 Australia	AD^b^: 25 HC: 123	Retinal vascular caliber; FD; Tortuosity; Branching of the retinal vascular network	AD: 72.4 (8) HC: 71.6 (6)	AD: 52% HC: 55%	Non‐mydriatic camera (Canon USA, Lake Success, NY, USA)	NA	AD showed a number of distinct retinal vascular parameters
Ong et al.,^117^	Observational case–control study *n* = 268 Chinese	CI‐mild: 78 CI‐moderate: 69 HC: 121	Retinal vascular caliber; FD; Tortuosity	CI‐mild: 71.1(6) CI‐moderate: 74.1 (5) HC: 67.3 (4)	CI‐mild: 54% CI‐moderate: 68% HC: 44%	Non‐mydriatic digital camera	Reduced fractal dimensions were associated with poorer cognitive performance	Reduced retinal vascular fractal dimensions linked to higher cognitive impairment risk
Williams et al.,^118^	Observational case–control study *n* = 507 UK	AD^b^: 213 HC: 294	Retinal vascular caliber; FD; Tortuosity; Branching of the retinal vascular network	AD: 79.6 (8) HC: 76.3 (7)	AD: 64% HC: 60%	500 Canon CR‐DGi digital camera	NA	AD: venular fractal dimension↓ and arteriolar tortuosity ↓
Qiu et al.,^119^	Observational case–control study *n* = 3906 USA	All: 3906	Retinopathy; Retinal vascular caliber	All: 76	All: 58%	Canon CR6 nonmydriatic digital camera	NA	The odds ratio of vascular dementia was 1.95 (1.04–3.62) for retinopathy
OCT retinal parameters correlated with cognitive function								
Pedersen et al.,^120^	Observational case–control study *n* = 134 Denmark	MCI^d^: 38 HC: 96	TMT mRNFL mGCL	MCI: 74.0 (70.0, 75.0) HC: 71.5 (68.5, 76.0)	MCI: 39% HC: 26%	Heidelberg Engineering Spectralis OCT Family (V6.9a)	NA	MCI: TMT↓ mRNFL↓ mGCL↓
Kim et al.,^121^	Longitudinal cohort study *n* = 430 Korea	NA	mRNFL;GCL; IPL; INL; OPL; ONL	All: 76.3 (7)	All: 48.6%	Heidelberg Engineering Spectralis OCT	BaselinemRNFL thickness correlated with baseline scores of CERAD and MMSE, as well as the decline in their follow‐up scores	The group with mRNFL thickness below the lowest quartile showed a much higher rate of MCI and AD prevalence than the group with thickness above the lowest quartile
Ko et al.,^122^	Longitudinal cohort study *n* = 32,038 UK	NA	RNFL	All: 57.3	All: 54%	Topcon 3D OCT 1000 Mk2	RNFL↓associated with cognitive performance↓	Participants with an RNFL thickness in the 2 thinnest quintiles were almost twice as likely to have at least 1 test score be worse at follow‐up cognitive testing
Mutlu et al.,^123^	Longitudinal cohort study *n* = 3289 Netherlands	Follow up: 86 dementia 68 AD^a^	RNFL; GC‐IPL	All: 68.9 (10)	All: 57%	Topcon OCT‐1000	NA	All dementia and AD: RNFL↓
Ward et al.,^124^	Observational case–control *n* = 2483 Germany	NA	pRNFL;mGCL	All: 54.3 (14)	All: 56%	Heidelberg Engineering Spectralis OCT	NA	pRNFL↓ mGCL ↓ related with global function ↓
Ueda et al.,^125^	Observational case–control *n* = 1078 Japan	All‐cause dementia: 61 AD^a^: 56	GC‐IPL;RNFL	All: 74 (6.5)	All: 58.5%	DRI‐OCT Triton	NA	The presence of all‐cause dementia and AD ↑ was related with GC‐IPL↓
Chua et al.,^126^	Observational case–control *n* = 225 Singapore	AD^a^: 62 MCI^d^: 108 HC: 55	GCC; GCL; IPL; GC‐IPL; mRNFL;cpRNFL	AD: 73.3 (9) MCI: 73.4 (6) HC: 71.0 (5)	AD: 63% MCI: 54% HC: 56%	Cirrus spectral domain‐OCT	NA	MCI/AD: cpRNF↓mGCC↓vs. HC
Ito et al.,^127^	Observational case–control *n* = 975 Japan	Dementia^a^: 38 MCI^g^: 324 HC: 613	mRNFL; ppRNF; GC‐IPL; GCC; TMT	Dementia: 77.2 (6) MCI: 74.2 (6) HC: 72.4 (5)	Dementia: 55% MCI: 49% HC: 60%	RS‐3000 Advance OCT	NA	TMT and GCC were associated with the presence of dementia
Castilla‐Martí L et al.,^128^	Observational case–control *n* = 1280 Spain	MCI‐AD: 196, MCI‐Va: 112, AD: 578, VaD: 93 HC: 301^a^	CT	MCI‐AD: 75.9 (7), MCI‐Va: 77.1(8), AD: 81.1 (7), VaD: 82.0 (6) HC: 66.6 (7)	MCI‐AD: 51, MCI‐Va: 50%, AD: 72%, VaD: 56% HC: 64%	DRI‐OCT Triton	No correlations were found between MMSE scores and CT	VaD: CT↑vs. HC MCI‐Va: CT↑vs. HC MCI‐AD: CT↑vs. HC
Ibrahim et al.,^129^	Observational case–control *n* = 357 Italy	AD and early AD: 44 MCI: 139 HC: 174^h^	pRNFL;RPE	AD and early AD: 82.2 (6) MCI: 80.7 (6) HC: 79.1 (1)	AD and early AD: 66% MCI: 45% HC: 66%	RTVue XR 100 Avanti spectral domain OCT	NA	AD and early AD: RPE↓ Vs. HC MCI: pRNFL↑vs. HC
Girbardt et al.,^130^	Observational case–control *n* = 6471 Germany	NA	cpRNFL	All: 55.5	All: 53.1%	Heidelberg Engineering Spectralis HRA + OCT	NA	cpRNFL↓ was correlated with ↓cognitive performance
Gao et al.,^131^	Observational case–control *n* = 100 China	CI: 36 SCD: 35 HC: 29	RNFL	CI: 66.3 (6) SCD: 65.4 (4) HC: 65.8 (4)	CI: 61% SCD: 80% HC: 59%	SVision Imaging VG200S SS‐OCT	NA	SCD: RNFL↓vs. HC
Galvin et al.,^132^	Observational case–control *n* = 100 USA	MCI: 66 Dementia: 43 HC: 27^a^	RNFL, GC‐IPL	MCI: 71.9 (9) Dementia: 76.9 (7) HC: 63.6 (10)	MCI: 50% Dementia: 37.2% HC: 74.1%	Zeiss Cirrus HD‐OCT	GC‐IPL correlated with memory, global cognitive performance	MCI: GC‐ IPL↓vs. HC AD: GC‐ IPL↓vs. HC and MCI
den Haan et al.,^133^	Observational case–control *n* = 142 Netherlands	AD^a^: 57 HC: 85	RNFL, GCL, IPL	AD: 65.0 (8) HC: 67.9 (9)	AD: 44% HC: 51%	Heidelberg Spectralis spectral domain OCT	NA	D: Retinal thickness∼vs HC
Ascaso, et al.,^134^	Observational case–control *n* = 80 Spain	AD^a^: 18 aMCI^i^: 21 HC: 41	RNFL; TMT; TMV	AD and aMCI: 72.1 (9) HC: 72.9 (8)	AD and aMCI: 54% HC: 51%	Zeiss STRATUS OCT 3	NA	AD: RNFL↓vs. HC and MCI; TMV↓vs. HC aMCI: RNFL↓vs. HC; TMV↑vs. HC
Mathew et al.,^135^	Observational case–control *n* = 75 USA	SCD: 26 MCI: 17 AD: 4 HC: 28^j^	RNFL	SCD: 71.0 (6) MCI: 73.8 (8) AD: 68.6 (12) HC: 70.5 (6)	SCD: 58% MCI: 41% AD: 35% HC: 79%	Zeiss Cirrus HD OCT software version 7.5	NA	SCD, MCI, AD: RNFL∼vs. HC
Santos et al.,^136^	Observational case–control *n* = 56 USA	Preclinical AD^k^: 15 HC: 41	mRNFL, ONL, IPL	All: 65.4 (17)	All: 63%	Heidelberg SPECTRALIS SD‐OCT	Preclinical mRNFL↓unrelated to episodic memory or problem‐solving performance	Preclinical AD: mRNFL↓, ONL↓, IPL↓vs. HC
Almeida et al.,^137^	Observational case–control *n* = 47 Brazil	MCI^l^: 23 HC: 24	mRNFL;GC‐IPL;GCC	MCI: 67.4 (7) HC: 64.6 (9)	MCI: 83% HC: 67%	DRI OCT Triton	MMSE and MoCA findings were significantly correlated with most macular thickness parameters	MCI: mRNFL↓, GC‐IPL↓, GCC↓vs. HC
Cheung et al.,^138^	Observational case–control *n* = 264 Singapore	AD^a^: 100 MCI^d^: 41 HC: 123	RNFL; GC‐IPL	AD: 73.5 (6) MCI: 70.4 (10) HC: 65.7 (4)	AD: 57% MCI: 68% HC: 46%	Zeiss CIRRUS SD‐OCT	NA	AD: RNFL↓GC‐IPL↓vs. HC MCI: GC‐IPL↓vs. HC
Burke et al.,^139^	Observational case–control *n* = 69 Singapore	Low risk (FH‐*APOE* ε4‐): 26; Medium risk (FH+ or *APOE* ε4+): 26; High risk (FH+ and *APOE* ε4+): 17	Choroidal measures (Total choroid area; Choroid thickness; Choroidal vascularity index; Total vessel area)	Low risk: 50.6 (6) Medium risk: 52.4 (4) High risk: 51.6 (6)	Low risk: 54% Medium risk: 65% High risk: 59%	Heidelberg spectral domain SPECTRALIS Retina HRA+OCT	NA	The presence of *APOE* ε4 and/or FH was significantly associated with larger total choroidal area and total vessel area
OCTA retinal parameters correlated with cognitive function								
Ma et al.,^140^	Observational case–control study *n* = 139 Spain	AD^a^: 43 MCI^a^: 62 HC: 34	VD; PD; FAZ	NA	AD: 47% MCI: 60% HC: 51%	Zeiss Cirrus 5000	VD & PD correlated significantly with MMSE and MoCA	MCI: VD↓PD↓, FAZ∼(vs. HC) AD: VD↓PD↓, FAZ∼ (vs. HC)
Bulut et al.,^141^	Observational case–control *n* = 52 Turkey	AD^a^, ^b^: 26 HC: 26	VD; FAZ; CT	HC: 72.6 (6.28) AD: 74.2 (7.55)	AD: 58% HC: 50%	RTVue XR Avanti	MMSE correlated positively with VD, CT, and negatively with FAZ	AD: VD & CT↓, FAZ↑ (vs. HC)
Criscuolo et al.,^142^	Observational case–control *n* = 56 Italy	aMCI^a^: 27 HC: 29	VD; FAZ	aMCI: 73 (6) HC: 73.1 (7)	aMCI: 56% HC: 52%	RTVue XR Avanti	No correlation with MMSE	SD‐aMCI: FAZ↑ MD‐aMCI: VD↓FAZ↑ (vs. HC)
Yoon et al.,^143^	Observational case–control *n* = 209 USA	AD^a^: 39 MCI^a^: 37 HC: 133	VD; PD; FAZ	AD: 72.8 (7.7) MCI: 71.1 (7.6) HC: 69.2 (7.8)	AD: 67% MCI: 54% HC: 73%	Zeiss Cirrus 5000	VD but not FAZ, was significantly correlated with MMSE	AD: VD↓PD↓(vs. HC and MCI), FAZ∼
Chua et al.,^144^	Observational case–control *n* = 90 Singapore	AD^b^: 24 MCI^d^: 37 HC: 29	VD; PD; FD; FAZ	AD: 74.9 (6) MCI: 77.9 (6) HC: 76.7 (5)	AD: 73% MCI: 44% HC: 45%	Zeiss Cirrus 5000	No correlation with MMSE	AD: VD↓FD↓(vs. HC) MCI: VD↓FD↓(vs. HC)
Wang et al.,^145^	Observational case–control *n* = 222 China	AD^a^: 77 HC: 145	VD; FAZ	AD: 61.9 ± 8.4 HC: 60.3 ± 7.1	AD: 69% HC: 61%	Zeiss Cirrus 5000	VD significantly correlated with MMSE scores	AD: VD↓(vs. HC) FAZ∼
Wang et al.,^146^	Observational case–control *n* = 158 China	AD^a^: 62 MCI^a^: 47 HC: 49	VD; FAZ	AD: 71.8 (8) MCI: 72.7 (8) HC: 69.5 (6)	AD: 56% MCI: 62% HC: 65%	RTVue XR Avanti	No significant correlation with MMSE score	AD: VD↓(vs. HC); FAZ∼ MCI: VD↓(vs. HC); FAZ∼
Marquié et al.,^147^	Observational case–control *n* = 672 Spain	HC: 128 MCI‐AD^a^, ^d^ :120 MCI‐VaD^a^, ^d^: 111 AD^a^: 257 VaD^a^: 56	VD	HC: 65.4 (7) MCI‐AD: 75.9 (7) MCI‐Va: 77.1 (7) AD: 80.4 (7) VaD: 80.5 (8)	HC: 70% MCI‐AD: 47% MCI‐Va: 52% AD: 70% VaD: 63%	DRI OCT Triton	MMSE not correlated to VD	MCI‐AD: VD↑(vs. HC) MCI‐Va: VD↓(vs. HC) AD or VaD: VD∼(vs. HC)
Biscetti et al.,^148^	Observational case–control *n* = 37 Italy	MCI‐AD^a^:24 HC: 13	VPD; VLD; FD	MCI‐AD: 72.1 (6) HC: 73.6 (4)	MCI‐AD: 63% HC: 54%	Heidelberg Engineering Spectralis HRA + OCT2	NA	MCI‐AD: VLD↓VPD↓FD↑(vs. HC)
García‐Sánchez et al.,^149^	Observational case–control *n* = 188 Spain	SCD^m^: 89 MCI^d^: 99	VD	SCD: 68.5 (7) MCI: 64.4 (7)	SCD: 66% MCI: 61%	DRI OCT Triton	NA	MCI: VD↑ Vs. SCD
Kwapong et al.,^150^	Observational case–control *n* = 157 China	EOAD^a^: 84 HC: 73	VD	Group1: (SS‐OCTA) EOAD: 62.2 (6) HC: 64.0 (6) Group2: (SD‐OCTA) HC: 58.0 (7) EOAD: 60.2 (7)	Group1: (SS‐OCTA) HC: 61% EOAD: 41% Group2: (SD‐OCTA) HC: 52% EOAD: 49%	SS‐OCTA: SVision Imaging VG200 SD‐OCTA: RTVue XR Avanti	NA	EOAD: VD↓(vs. HC)
López‐Cuenca et al.,^151^	Observational case–control *n* = 83 Spain	FH‐ *APOE* ε4‐: 18 FH‐ *APOE* ε4+: 3 FH+ *APOE* ε4‐: 42 FH+ *APOE* ε4+: 20^m^	VD	All: 45‐80	NA	Heidelberg Engineering Spectralis II	NA	Macular analysis: FH+ ApoE E4+: VD↓vs. FH+ ApoE E4‐ Peripapillary analysis: FH+ ApoE E4+: VD↑vs. FH‐ ApoE E4‐ and FH+ ApoE E4‐
Shin et al.,^152^	Observational case–control *n* = 55 Korean	MCI‐AD^a^:24 HC: 31	VD; FAZ	MCI‐AD: 72.8 (9) HC: 69.0 (10)	MCI‐AD:63% HC: 65%	Zeiss Cirrus 5000	NA	MCI‐AD: VD↓FAZ↑(vs. HC)
Wu et al.,^153^	Observational case–control *n* = 64 China	AD^b^: 18 MCI^d^: 21 HC: 21	VD; FAZ	AD: 69.9 (6) MCI: 67.8 (6) HC: 67.8 (6)	AD: 50% MCI: 43% HC: 48%	RTVue XR Avanti	NA	AD: VD↓FAZ↑(vs. HC and MCI) MCI: VD↓FAZ↑(vs. HC)
Xie et al.,^154^	Observational case–control *n* = 158 China	AD^a^: 55 MCI^d^: 41 HC: 62	VD; VLD;VFD; FAZ;FR	AD: 60.6 (6) MCI: 61.7 (8) HC: 58.7 (7)	AD: 58% MCI: 34% HC: 47%	RTVue XR Avanti	NA	AD: VD↓VLD↓(vs. HC) MCI: VD↓VLD↓VFD↓(vs. HC) FR↑(vs. HC)
O'Bryhim et al.,^155^	Observational case–control *n* = 30 USA	Preclinical AD^e^: 14 HC: 16	VD; FAZ	Preclinical AD: 73.5 (4) HC: 75.2 (7)	Preclinical AD: 43% HC: 62%	Avanti Optovue OCTA	NA	Preclinical AD: FAZ↑(vs. HC)
van de Kreeke et al.,^156^	Observational case–control *n* = 124 Netherlands	Preclinical AD (Aβ+): HC: 111	VD; FAZ	All: 53.2%	All: 53.2%	Zeiss Cirrus 5000	NA	Preclinical AD: VD↑FAZ∼(vs. HC)
Ashimatey et al.,^157^	Observational case–control *n* = 61 USA	Cognitively normal: 35 Cognitively abnormal^o^:15 Not defined:11	VSD	All: 68 (6)	All: 79%	Zeiss HD‐OCTA	A 0.01‐unit lower VSD raises 20% risk of CDR‐SOB > 0, links to lower total MoCA, strongest with its Visuospatial/Executive domain	VSD↓associated with abnormalities in cerebrovascular perfusion and function
Abdolahi et al.,^158^	Observational case–control *n* = 101 USA	NA	VD;VSD;VAF;VSF	All: 66.4 (10)	All: 68%	Zeiss Meditec PLEX Elite 9000	VD↓and VAF↓correlated with Oral Symbol Digit Test ↓and Fluid Cognition scores↓	Retinal metrics (VD, VAF, VSF) associated with sensitive biomarkers of small vessel VCID from MRI
Abraham et al.,^159^	Longitudinal cohort study *N* = 976 USA	NA	VD; FAZ	All: 78	All: 62%	RTVue XR Avanti	No associations of VD or FAZ with longitudinal changes in either global cognitive function	No VD/FAZ links to Incident MCI/dementia
O'Bryhim et al.,^160^	Longitudinal cohort study *n* = 20(3‐ year follow‐up) USA	biomarker negative: 11 biomarker positive: 9^e^	FAZ	biomarker negative: 75.2 (4) biomarker positive: 76.3 (5)	NA	Optovue OCTA	NA	Biomarker positive: FAZ↑vs. biomarker negative (baseline and 3‐year follow‐up)
Hu et al.,^161^	Observational case–control *n* = 52 China	CI^p^: 31 HC: 21	VD; FAZ	CI: 67.0 (8) HC: 61.4 (8)	CI: 71% HC: 67%	Zeiss Cirrus 5000	NA	CI: VD↓vs. HC
Lee et al.,^162^	Observational case–control *n* = 60 Korea	ADCI: 28 SVCI: 18 HC: 14^q^	CD	ADCI: 67.5 (10) SVCI: 77.0 (6) HC: 67.2 (6)	ADCI: 61% SVCI: 67% HC: 71%	DRI OCT Triton plus	NA	SVCI: CD↓vs. HC (the temporal quadrant of the RPC network) SVCI: CD↓vs. ADCI (superior quadrant and the temporal quadrant of the RPC network)

Abbreviations: AD, Alzheimer's disease; aMCI, amnestic mild cognitive impairment; *APOE*, apolipoprotein E; CASI‐S, Cognitive Abilities Screening Instrument‐Short Form; CD, capillary density; CERAD, Consortium to Establish a Registry for Alzheimer's Disease score; CFP, color fundus photography; CI, cognitive Impairment; cpRNFL, circumpapillary retinal nerve fiber layer; CRAE, central retinal arteriolar equivalent; CRVE, central retinal venular equivalent; CT, choroid thickness; DSS, digit symbol substitution; DSST, digit symbol substitution test; FAZ, foveal avascular zone; FD, fractal dimension; FH, family history; FR, roundness of the FAZ; GCC, ganglion cell complex; GCL, ganglion cell layer; GC‐IPL, ganglion cell‐inner plexiform layer; HC, healthy control; INL, inner nuclear layer; IPL, Inner plexiform layer ; MCI, mild cognitive impairment; MCI‐AD, mild cognitive impairment due to Alzheimer's; MCI‐Va, mild cognitive impairment due to cerebrovascular disease; MD, multiple domains; mGCC, macular ganglion cell complex; mGC‐IPL, macular ganglion cell‐inner plexiform layer; mGCL, macular ganglion cell layer; MMSE, Mini‐mental state examination; mRNFL, macular retinal nerve fiber layer; OCT, optical coherence tomography; OCTA, optical coherence tomography angiography; ONL, outer nuclear layer; OPL, outer plexiform layer; PD, blood perfusion density; ppRNFL, peripapillary retinal nerve fiber layer; RNFL, retinal nerve fiber layer; RPC, radial peripapillary capillary; RPE, retinal pigment epithelium; SCD, subjective cognitive decline; SD, single domain; TMT, total macular thickness; TMV, total macular volume; VaD, vascular dementia; VAF, vessel area flux; VD, vessel density (vessel area density); VFD, vascular fractal dimension; VLD, vessel length density; VPD, vascular perfusion density; VSD, vessel skeleton density; VSF, vessel skeleton flux; WF, word fluency test.

^a^ Vascular dementia (VaD) was diagnosed based on NINDS‐AIREN International Workshop Criteria.^163^ Alzheimer's disease dementia (AD) was defined using NIA‐AA criteria.^164^ MCI‐AD group was characterized by memory impairment and the absence of other comorbidities that could explain the cognitive decline (probable amnestic MCI) with suspected underlying AD. The MCI‐Va group was defined based on the suspected underlying etiology of cerebrovascular pathology.

^b^ The dementia syndrome was diagnosed using the Diagnostic and Statistical Manual of Mental Disorders, 4th edition (DSM‐IV) criteria, and the diagnosis of AD followed the National Institute of Neurological Disorders and Stroke (NINDS)–Alzheimer Disease and Related Disorders Association (ADRDA) criteria.^165^ ^.^

^c^ MCI defined by a Montreal Cognitive Assessment (MoCA) score ≤ 26, with subjective cognitive decline, in the absence of depression or problems with activities of daily living.

^d^ Petersen's Individuals were considered to have MCI if the average score one or more domains was below the 15th percentile or if more than half of the test scores in a single domain were below the 5th percentile.^166^ ^.^

^e^ Preclinical AD: PET imaging for PiB or 18F‐AV‐45 compound or CSF analysis of Aβ42 protein level.

^f^ The diagnoses of dementia and AD were made in accordance with internationally accepted criteria for dementia (DSM‐III‐R).

^g^ Participants who were not demented but had clinically significant memory impairment were categorized as having MCI.

^h^ (1) HCs: maximum scores in both assessments or no more than −2 points of selective deficit; (2) MCI: cognitive impairment was confirmed by deficient scores in one or more cognitive domains of MMSE or FAB (performance between −1 and −2 standard deviations); (3) AD and early AD (eAD) denoted as “Dem”: deficit scores in all cognitive domains (performances −2 standard deviations).

^i^ Winblad et al. criteria of mild cognitive impairment—beyond controversies, toward a consensus: report of the International Working Group on Mild Cognitive Impairment.^167^ ^.^

^j^ SCD participants had subjective impairment of cognition as measured using the Cognitive Change Index (CCI) and no impairment on cognitive testing; MCI patients were functioning independently but had concerns about cognition from themselves, an informant, and/or a clinician and evidence of impaired cognitive testing (> −1.5 standard deviations) in one or more cognitive domains (most commonly memory); AD patients had an impaired ability to function independently at work or usual activities with evidence of impaired cognitive testing in one or more domains.

^k^ Preclinical‐stage disease based on evidence of both (1) elevated neocortical Ab burden as determined by PET amyloid imaging and (2) relative cognitive impairments in response to a challenge with a very low–dose muscarinic anticholinergics.^168^ ^.^

^l^ An analysis of systems of classifying mild cognitive impairment in older people.^169^ ^.^

^m^ SCD: A conceptual framework for research on subjective cognitive decline in preclinical Alzheimer's disease.^170^ ^.^

^n^ The (FH+) group consisted of subjects with at least one parent with sporadic AD. These subjects were not required to have a history of neurological or psychiatric disorders or to have severe disease.

^o^ Clinical status was defined according to the standard guidelines stipulated in the 3rd version of the Uniform Data Set used in the National Alzheimer's Coordinating Center (NACC).^171^ ^.^

^p^ The MoCA was used to identify HC and CI. Optimal cutoff points were determined based on education level. For individuals with 7 or more years of education, the MoCA cutoff was 24/25. For those with 1–6 years of education, the MoCA cutoff was 19/20. Finally, for those with no formal education, the cutoff was 13/14.

^q^ (1) ADCI comprises AD dementia and amnestic mild cognitive impairment (aMCI) due to AD, which was diagnosed based on the National Institute on Aging–Alzheimer's Association research criteria for probable AD dementia and aMCI due to AD, respectively; (2) SVCI was diagnosed based on the following criteria: (i) subjective cognitive complaint by the patient or caregiver; (ii) objective cognitive impairment less than the 16th percentile of the age‐ and education‐matched norm in any domain including language, visuospatial, memory, or frontal function on neuropsychological tests; (iii) presence of severe ischemia on brain MRI; and (iv) focal neurologic symptoms or signs.

#### 2.3.1 Color fundus photography (CFP)

CFP offers both qualitative and quantitative retinal biomarkers associated with cognitive impairment^34^, ^172^ (Table 2). Qualitative retinal parameters such as microaneurysms, hemorrhages, and cotton wool spots are associated with cognitive dysfunction.^88^, ^103^, ^108^ Longitudinal evidence including the Atherosclerosis Risk in Communities (ARIC) study also links retinopathy to accelerated cognitive decline.^109^, ^110^ Nevertheless, research findings across studies remain inconclusive regarding this association. The Rotterdam Study identified an association between retinopathy and prevalent dementia (adjusted OR = 2.04, 95% CI: 1.34–3.09), although no association was observed with incident dementia or its subtypes after a mean 11.4‐year follow‐up (adjusted HR = 1.15, 95% CI: 0.88–1.48).^105^ Subsequent studies specifically indicate that retinopathy manifestations such as retinal hemorrhages and arteriovenous nicking may predict cerebrovascular‐related dementia or MCI, though not primary AD.^111^, ^112^ Similarly, the Cardiovascular Health Study suggested that retinopathy and focal arteriolar narrowing elevated dementia risk in hypertensive individuals (OR = 2.10–3.02).^102^ Consistent with this pattern, the AGES‐Reykjavik Study identified an association between retinopathy and VD (OR = 1.95).^119^ Such subtype‐specific differences may arise from variations in underlying neuropathology.^173^ However, several studies failed to identify significant associations: both the Northern Ireland Cohort for the Longitudinal Study of Ageing (NICOLA) and the Los Angeles Latino Eye Study (LALES) reported no significant associations between retinal changes and cognitive dysfunction in their respective populations.^100^, ^114^ Such heterogeneity may stem from differences in study populations (e.g., age distribution, comorbidity profiles) and variability in retinal sign assessment criteria.

Quantitative retinal parameters offer a more refined approach to assessing cognitive risk. Key parameters include central retinal arteriolar equivalent (CRAE) and wider central retinal venular equivalent (CRVE), with narrower arterioles and wider venules predicting cognitive decline (HR = 1.26 and 1.20 per standard deviation change).^113^, ^174^ Generalized arteriolar narrowing corresponds to elevated disabling dementia risk (crude OR = 1.66, 95% CI: 1.19–2.31).^107^ A prospective deep‐learning study confirmed these associations and further linked narrower arterioles to incident dementia in cognitively impaired individuals.^115^ However, these associations are modified by sex and dementia subtype. Sex‐specific differences further complicated interpretations, with retinal calibers showing stronger associations with verbal memory deficits in males.^101^ The Rotterdam Study further highlighted larger venular calibers were linked with increased VaD risk (HR = 1.31 per SD; 95% CI: 1.06–1.64) but not AD.^104^ Additional biomarkers show relevance: Reduced fractal dimension (FD), reflecting a sparser microvascular network, has been associated with both preclinical dementia^117^ and AD,^118^ particularly correlating with cerebral Aβ and tau burden.^175^ Increased venular tortuosity, linked to blood viscosity and vascular dysfunction,^176^, ^177^ also correlated with AD risk.^106^ Advanced imaging studies have identified peripheral biomarkers such as drusen and venular widening in AD patients,^178^ while features like branching asymmetry and arteriolar length‐to‐diameter ratio have distinguished individuals with high cerebral plaque burden.^116^ These retinal alterations reflect corresponding cerebral microvascular pathology, suggesting shared underlying mechanisms.^118^

Despite its potential, CFP has limitations. Imaging primarily focuses on the central retina, potentially missing peripheral abnormalities.^179^ Qualitative assessments exhibit observer variability, while image quality may be compromised by anatomical and technical factors,^180^ with both aspects subject to confounding influences including cardiovascular risk factors. Moreover, quantitative parameters such as FD lack standardization across studies, limiting their comparability. Semi‐automated analysis tools improve reproducibility, but broader validation across diverse populations, incorporation of more longitudinal studies and integration with advanced imaging modalities are needed for clinical translation.^110^

#### 2.3.2 Optical coherence tomography (OCT)

OCT is a high‐resolution imaging technique based on interferometric principles that enables detailed visualization and quantification of the retinal layers. This makes it a valuable tool for investigating retinal biomarkers associated with cognitive decline (Table 2). The retina consists of ten distinct layers, which are key central nervous system neurons located in the retina, have their cell bodies in the ganglion cell layer (GCL), dendrites in the inner plexiform layer (IPL), and axons in the retinal nerve fiber layer (RNFL); these axons form the optic nerve and transmit visual information to the brain.^35^

Previous studies have shown that retinal layer thickness varies across different stages of cognitive impairment, potentially reflecting underlying pathological processes at each stage. Individuals with subjective cognitive decline (SCD) exhibit significant thinning of the peripapillary RNFL (pRNFL) compared to cognitively normal controls. Moreover, a positive correlation has been observed between macular ganglion cell complex (mGCC) thickness and cerebral blood flow in SCD, suggesting that early retinal neuronal alterations may parallel initial brain changes along the AD continuum.^131^ In the preclinical stage of AD, longitudinal studies have reported decreased thickness of the inner fovea, outer nuclear layer (ONL), and IPL, with progressive macular RNFL (mRNFL) thinning**—**which is associated with amyloid burden and subtle functional changes preceding overt cognitive symptoms.^136^, ^155^ Notably, research on cognitively healthy individuals at elevated AD risk, such as apolipoprotein E (*APOE*) ε4 carriers and those with familial AD history, shows increased CT and larger choroidal vascular indices. Such vascular alterations may reflect an early vascular response or a link to AD risk factors prior to measurable cognitive decline.^139^ However, no significant changes in retinal thickness were found in some studies, due to differences in the diagnosis and age of the population, as this subset of MCI individuals was older (over 70 years of age).^152^

Within the MCI population, diffuse thinning of inner retinal layers is observed across multiple parameters, including reductions in macular ganglion cell‐IPL (GC‐IPL) thickness,^138^ mGCC thickness,^126^, ^137^ macular GCL thickness,^120^ and mRNFL^90^, ^121^, ^122^; peripapillary RNFL (pRNFL) thinning has also been documented.^126^, ^129^, ^134^ Furthermore, in cerebrovascular‐related MCI (MCI‐Va), increased CT may differentiate patients from cognitively unimpaired controls and sometimes from AD‐spectrum MCI.^128^ AD patients also exhibit a similar pattern of inner macular layer thinning, including progressive reductions in the GC‐IPL,^125^, ^132^, ^138^ GCC,^126^, ^127^ mRNFL,^138^ and full macular thickness.^127^ Thinning of the pRNFL is also evident,^126^, ^129^, ^134^ with bilateral pRNFL thickness positively correlating with brain volumes.^135^ Longitudinal population‐based studies have shown that thinner baseline pRNFL is associated with a higher risk of developing both all‐cause dementia and AD dementia.^123^ Additionally, macular thickness and GC‐IPL thickness have been correlated with the volumes of cognitively relevant brain regions such as the hippocampus and entorhinal cortex.^125^, ^132^, ^133^ It should be noted that a large amyloid‐confirmed AD cohort study found no significant difference in overall retinal thickness between patients and controls,^133^ suggesting methodological variations may influence retinal biomarker detection.

Unlike AD, vascular cognitive impairment (VCI) shows no significant differences in RNFL thickness compared to cognitively normal controls or AD groups.^162^ In contrast, VaD is associated with significantly increased CT, particularly in peripheral macular regions. This pattern resembles findings in MCI‐Va and is distinct from the thinning patterns typically seen in AD,^128^ indicating that CT may help differentiate AD from cardiovascular‐related pathogenic pathways. In addition, structural OCT parameters also show associations with dementia risk and cognitive function. Population‐based studies report that thinner baseline RNFL thickness correlates with poorer baseline cognitive performance^130^ and predicts future cognitive decline.^121^, ^122^ Similarly, reduced mGCL thickness is linked to lower global cognitive function, with this association modulated by age and vascular risk factors.^124^

**FIGURE 1:**
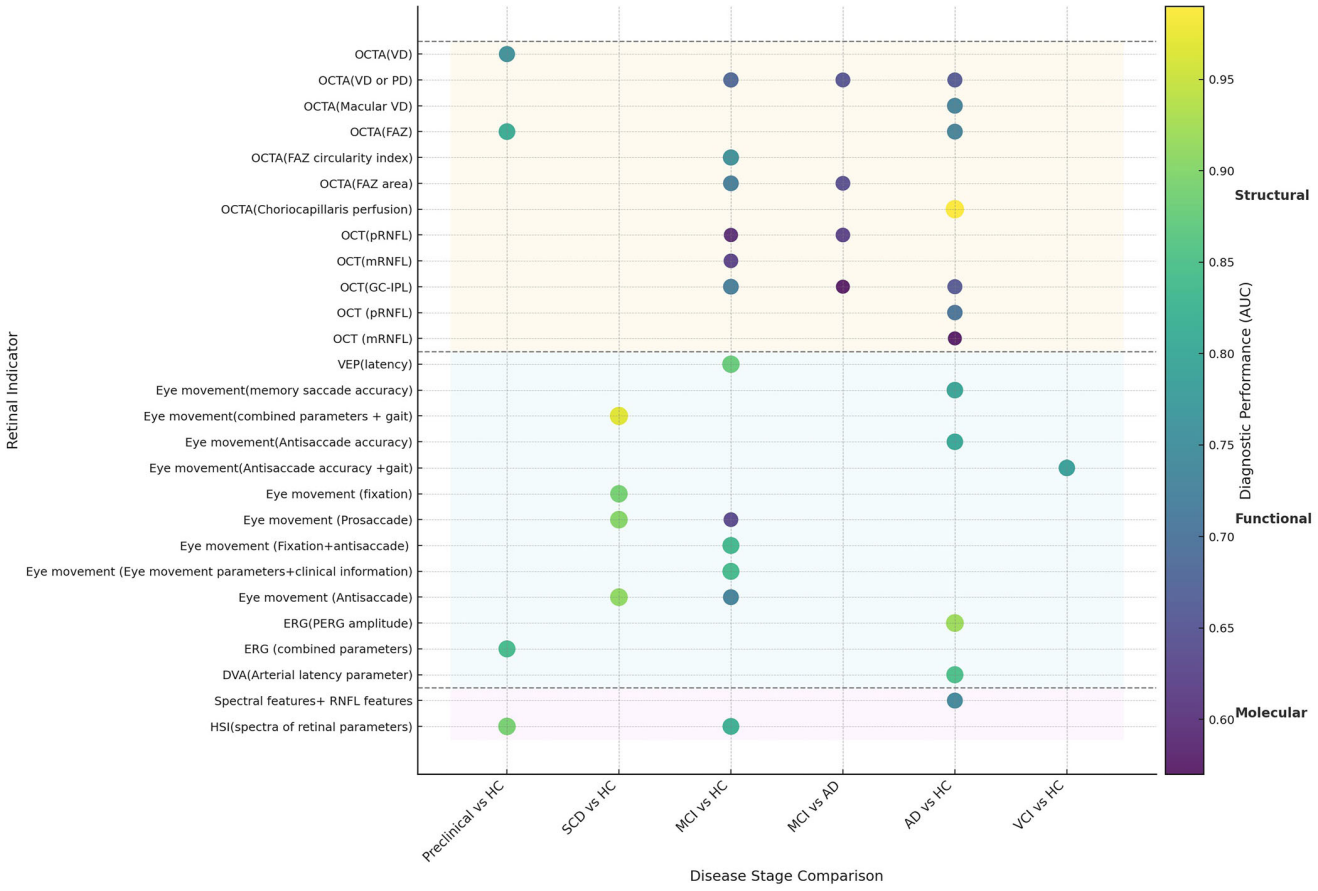
Diagnostic performance of retinal biomarkers across disease stages.

The discriminative ability of OCT parameters varies by cognitive comparison group, retinal layer, and analytical method (Figure 1). For distinguishing AD from healthy controls (HC), GC‐IPL thickness exhibits moderate diagnostic performance (area under the curve [AUC] = 0.63–0.69),^37^, ^38^, ^138^ while mRNFL thickness shows lower discriminative ability (AUC = 0.57, 95% CI: 0.50–0.64) and pRNFL higher classifying efficacy (AUC = 0.70, 95% CI: 0.53–0.79).^37^ In comparisons between MCI and HC, discriminative performance is generally reduced: pRNFL achieves an AUC of 0.59,^17^ mRNFL shows weaker results (AUC = 0.58–0.62),^16^, ^37^ and GC‐IPL thickness yields variable AUC values (0.55–0.72).^37^, ^38^, ^138^, ^181^ When distinguishing MCI from AD, GC‐IPL thickness exhibits moderate discriminative performance (AUC = 0.57–0.82) by meta‐analyses.^37^, ^132^ pRNFL thickness shows AUC values of 0.54–0.62,^36^, ^37^ with algorithm‐compensated RNFL (AUC = 0.74) outperforming measured RNFL (AUC = 0.69).^126^ Combined macular and compensated pRNFL parameters reach the highest discriminative ability (AUC = 0.80).^126^

Emerging approaches enhance diagnostics, as OCT‐intensity spatial correlation features achieve higher accuracy for detecting MCI (AUC = 0.830) and AD (AUC = 0.935) than conventional thickness measures (AUC = 0.705–0.795).^182^ In summary, GC‐IPL and pRNFL have moderate predictive power in distinguishing MCI and AD from healthy individuals, while the discriminative ability of other layer thicknesses is generally limited, with existing heterogeneity (Figure 1). Heterogeneity in discriminative ability stems from methodological variations including study design, sample characteristics (e.g., ethnicity, age, comorbidities), OCT device/segmentation protocols, and anatomical differences–trilateral models show better performance in Asian versus White cohorts due to anatomical variation and model optimization.^183^ Although composite parameters show relatively good discriminative ability, they require further validation and replication.

In conclusion, macular and peripapillary RNFL undergo progressive thinning from SCD stages through MCI and AD, potentially reflecting early cerebral alterations**—**though validation against neuropathological parameters and expanded SCD research remain needed. While GC‐IPL, GCC, mRNFL, and pRNFL changes occur in both MCI and AD, their discriminative efficacy is modest. CT may differentiate VCI from AD‐associated cognitive impairment (ADCI) pathologies, yet no studies currently utilize CT for disease differentiation, indicating a critical research gap. Key limitations include predominance of cross‐sectional designs, limited longitudinal follow‐up, and insufficient large‐scale studies, restricting causal inference regarding retinal‐cognitive temporal relationships. Inconsistent OCT segmentation protocols^133^, ^134^ and inadequate adjustment for confounders (e.g., age, cardiovascular risks, axial length)^124^, ^128^, ^139^ further constrain clinical translation. Future priorities comprise large longitudinal cohorts employing standardized OCT analytical pipelines, diverse population sampling, and rigorous confounder adjustment to establish clinically viable biomarkers.

#### 2.3.3 Optical coherence tomography angiography (OCTA)

OCTA enables visualization and quantification of the retinal microvasculature without contrast dye injection,^144^, ^155^ offering potential for detecting changes associated with different clinical stages and types of cognitive impairment (Table 2).

In preclinical AD, amyloid‐positive individuals consistently exhibit foveal avascular zone (FAZ) enlargement, suggesting early capillary dropout,^155^ though paradoxically increased vessel density (VD) in macular subregions may indicate vascular compensation.^151^, ^156^ MCI presents more pronounced microvascular abnormalities with etiological distinctions. AD‐spectrum MCI (MCI‐AD) shows reduced VD in both deep and superficial capillary plexuses (DCP/SCP), particularly in temporal/inferior quadrants,^129^, ^142^, ^146^, ^153^ with exacerbated DCP deficits in *APOE* ε4 carriers.^152^ In one study, MCI‐AD exhibited higher VD in the temporal quadrant, while MCI‐Va showed preferential inferior quadrant VD reduction, suggesting distinct microvascular pathological profiles between these subtypes.^147^ Further studies are required to validate these differences. Additionally, MCI cohorts show lower perfusion density (PD)^140^ and altered FD reduced in SCP but elevated in DCP, suggesting divergent pathophysiology.^148^ Additional findings include decreased vascular length densities, increased vascular tortuosity and increased FAZ circularity,^154^ though some studies report no FAZ differences across groups^144^, ^146^ or longitudinal VD/FAZ‐cognition associations.^159^, ^160^

AD manifests widespread retinal microvascular loss, featuring consistent VD/PD reductions in SCP/DCP,^143^, ^146^ with most severe in nasal/inferior subregions,^140^, ^145^ suggesting neurovascular uncoupling.^161^ These impairments correlate with cognitive measures (e.g., MMSE, Montreal Cognitive Assessment [MoCA], visuospatial function, executive function).^140^, ^141^ Early‐onset AD shows choriocapillaris attenuation correlating with CSF Aβ42/tau and serum p‐tau181.^150^ While FAZ enlargement is frequently reported,^141^ methodological variability may account for discrepant results in studies reporting no significant FAZ area differences.^143^, ^144^, ^146^ Crucially, retinal parameters may reflect cerebral structural changes: nasal macular VD associates with hippocampal volume independent of amyloid status,^149^ and reduced VD correlates with subiculum/presubiculum atrophy in cognitive impairment.^161^

VCI exhibits distinct OCTA patterns: significantly reduced capillary density (CD) in the temporal quadrant compared to cognitively normal controls, with additional reductions in superior/temporal quadrants relative to ADCI.^162^ Unlike AD, superficial plexus VD shows no consistent correlation with hippocampal atrophy in VCI.^149^ Notably, impaired retinal capillary perfusion correlates with cognitive deficits, cerebrovascular hypoperfusion, and MRI biomarkers of cerebral small vessel disease (CSVD),^158^ supporting OCTA's utility in identifying retinal correlates of VCI‐associated neurovascular abnormalities.^157^

OCTA parameters exhibit variable diagnostic performance across the cognitive impairment spectrum (Figure 1). In preclinical AD, FAZ enlargement differentiates biomarker‐positive individuals from HC with an AUC of 0.81,^155^ while VD around the optic nerve head yields an AUC of 0.76.^156^ To distinguish MCI from HC, meta‐analyses report that VD or PD in DCP and SCP achieve AUC values of 0.50–0.68,^37^ whereas new composite parameters show superior performance: multi‐view modules (AUC = 0.88),^184^ full‐width‐at‐half‐maximum VD (AUC = 0.84),^185^ and FAZ circularity (AUC = 0.76).^186^ Differentiation of AD from HC showed high diagnostic ability for choriocapillaris perfusion (AUC = 0.91–0.99).^150^ Artificial intelligence (AI) ‐enhanced multi‐view models achieved an AUC of 0.89,^184^ whereas macular VD‐based models yielded an AUC of 0.73.^145^ Recent reviews report that VD or PD in the DCP achieves AUC values of 0.55 (95% CI: 0.50–0.61) and 0.64–0.66 in the SCP,^37^, ^187^ while FAZ area contributes an AUC of 0.73 (0.50–0.89).^37^

Cross‐stage discrimination (AD/MCI vs. HC) shows moderate capacity for superficial FD (AUC = 0.77), superficial VD (AUC = 0.72), and deep VD (AUC = 0.64).^144^ SCD and VCI evidence remains limited without robust diagnostic parameters. Performance variability stems from OCTA platforms,^150^ sample heterogeneity, and methodological disparities between conventional analyses and AI‐based models,^184^, ^185^ necessitating standardization for clinical translation.

Collectively, VD and FAZ parameters undergo early changes in cognitive decline: VD may temporarily increase in preclinical stages but decreases in MCI and AD, while FAZ consistently enlarges across all stages. Both parameters demonstrate moderate diagnostic accuracy (AUC > 0.7) for distinguishing preclinical stages, MCI, and AD from HC and correlate with structural neuroimaging markers like hippocampal atrophy. PD declines in MCI and AD, and CD reductions may help distinguish AD from VaD, though further validation is needed. Notably, choriocapillaris perfusion shows high diagnostic potential (AUC > 0.8) and neuropathological associations, as do certain AI‐derived composite parameters, but evidence for these remains limited. Current research is constrained by small sample sizes and cross‐sectional designs, limiting causal inference and progression tracking.^140^, ^142^ Establishing OCTA as a clinically viable biomarker requires standardized protocols, longitudinal validation against neuroimaging and fluid biomarkers,^187^ and expanded studies in underrepresented populations (e.g., SCD).

### 2.4 Parameters of fundus functional examination

Fundus functional examination parameters, including those from DVA, ERG, retinal oximetry, VEPs, and eye movement measurements, show potential correlations with cognitive function, aiding disease detection and mechanistic understanding (Table 3).

**TABLE 3:** Key studies on the association between retinal functional biomarkers and cognitive function.

Author	Cohort information	Sample	Retinal parameter	Age	Gender character (Female, %)	Retinal imaging	Cognitive outcome	Summary of results
Dynamic vessel analysis (DVA) parameters correlated with cognitive function								
Kotliar et al.,^188^	Observational case–control study *n* = 55 Germany	AD^a^: 15 MCI^a^: 24 HC: 16	Arterial and venous dilation and response	AD: 72.9 (9) MCI: 68.2 (9) HC: 66.4 (8)	AD: 60% MCI: 58% HC: 63%	DVAlight, IMEDOS Systems	NA	AD: Arterial and venous dilation↑arterial time to reach 30% of max dilation ↑vs. MCI and HC MCI: Arterial constriction ↑ vs. HC
Querques et al.,^189^	Observational case–control study *n* = 55 Italy	AD^a^: 12 MCI^a^: 12 HC: 32	Arterial and venous dilation; arterial constriction; reaction amplitude	AD: 72.9 (7) MCI: 76.3 (7) HC: 71.6 (6)	AD: 67% MCI: 58% HC: 47%	DVA; Imedos Systems UG, Jena, Germany	NA	AD: arterial dilation↓ reaction amplitude↓ vs HC MCI: reaction amplitude↓ vs. HC
Mroczkowska et al.,^190^	Observational case–control study *n* = 90 UK	AD^b^: 10 HC: 28	Reaction time: time that it takes retinal vessels to reach a maximum dilation	AD: 62.5 (8) HC: 57.9 (7)	AD: 50% HC: 39%	IMEDOS GmbH, Jena, Germany	Negative correlation between the arterial reaction time of the first cycle and MMSE score in patient	AD: Artery reaction time: Flicker 1 and 3↑Flicker 2↓
Electroretinography (ERG) parameters correlated with cognitive function								
Asanad et al.,^191^	Observational case–control study *n* = 90 Austria	Preclinical AD^c^: 15 HC: 14	P50, N95, a‐wave, b‐wave, PhNR, amplitude and implicit time	Preclinical AD: 76.5 (7) HC: 75.9 (9)	Preclinical AD: 80% HC: 71%	DTL‐Plus (Diagnosys LLC, Lowell, MA, USA)	NA	Preclinical AD: PhNR amplitudes↓ ffERG a/b wave implicit times ↑vs. HC
Katz et al.,^192^	Observational case–control study *n* = 12 USA	AD^b^: 6 HC: 6	PERG: a and b wave amplitude and latency	AD: 69.7 (4) HC: 67.2 (5)	AD: 33% HC: 33%	Nicolet Compact‐Four, Nicolet, Madison, WI	NA	AD: b wave amplitude↓ vs. HC
Krasodomska et al.,^193^	Observational case–control study *n* = 60 Poland	AD^b^: 30 HC: 30	PERG: amplitude and time of the P50 and N95 waves	AD: 72.9 (7)	AD: 67%	Roland Consult	NA	AD: P50 wave implicit time↑; P50 and N95 wave amplitudes ↓
Cabrera DeBuc et al.,^194^	Observational case–control study *n* = 39 USA	CI^d^: 20 HC: 19	Amplitude; Implicit time	CI: 81 (6) HC: 80 (7)	CI: 80% HC: 84%	RETevalTM, LKC Technologies, Inc., Gaithersburg, MD, United States)	NA	CI: Amplitude↓; Implicit time↑
Sen et al.,^195^	Observational case–control study *n* = 60 India	AD^b^: 20 HC: 40	mfERG: Amplitude; Implicit time	AD: 61.5 (7) HC: 60.9 (8)	–	Metrovision, Monpack, Pirenchies, France	NA	AD: P1, N1, and N2 amplitude↓; P1 implicit time↑
Retinal oximetry parameters correlated with cognitive function								
Szegedi et al.,^196^	Observational case–control study *n* = 90 Austria	AD^b^: 23 MCI^b^: 24 HC: 43	Oxygen saturation	AD and MCI: 73.4 (9) HC: 70.7 (7)	AD and MCI: 40% HC: 53%	DVA, Imedos, Jena, Germany	NA	AD and MCI: arteriovenous difference in oxygen saturation↓ vs. HC
Olafsdottir et al.,^197^	Observational case–control study *n* = 84 Iceland	MCI^e^: 42 HC: 42	Oxygen saturation	MCI: 70 (10) HC: 66 (9)	–	Oxymap ehf., Reykjavik, Iceland	NA	MCI: oxygen saturation↓ vs. HC
Einarsdottir et al.,^198^	Observational case–control study *n* = 84 Iceland	Mild AD^f^: 8 Moderate AD^a^: 10 HC: 18	Oxygen saturation	Mild AD: 65 (9) Moderate AD: 72 (4) HC: 64 (7)	Mild AD: 38% Moderate AD: 50% HC: 56%	Oxymap, ehf., Reykjavik, Iceland	NA	Moderate AD: oxygen saturation in arterioles and venules↓ vs. HC
VEP parameters correlated with cognitive function								
Stothart et al.,^199^	Observational case–control study *n* = 71 UK	AD^b^: 20 MCI^b^: 25 HC: 26	Visual P1, N1, and visual mismatch negativity (vMMN)	AD: 79.2 (9) MCI: 77.3 (7) HC: 76.0 (7)	AD: 65% MCI: 36% HC: 46%	BrainAmp DC amplifier (Brain Products GmbH)	P1 amplitude and vMMN amplitude were associated with MMSE	AD: P1 and N1 amplitudes↓vs. HC MCI: N1 amplitude↓ vs. HC
Yamasaki et al.,^200^	Observational case–control study *n* = 45 Japan	MCI^g^: 15 Old: 15 Young: 15	VEP latency	MCI: 74.4 (4) Old: 73.5 (5) Young: 27.9 (5)	MCI: 67% Old: 67% Young: 67%	Electrical Geodesics Inc., Eugene, Oregon	VEPs for higher‐dorsal stimuli were related to outcomes of neuropsychological tests	MCI: latency↑ vs. HC
Wang et al.,^201^	Observational case–control study *n* = 158 China	AD^a^: 47 MCI^a^: 52 HC: 51	VEP latency and amplitude	AD: 71.9 (5) MCI: 70.3 (7) HC: 69.3 (7)	AD: 68% MCI: 63% HC: 53%	32 channel elastic electrocap (g.Nautilus Research, g.tec, Austria)	NA	AD: N2 and P3 latencies↑P3 amplitudes ↓ vs. HC and MCI. MCI: P3 latencies↑ vs. HC
Fix et al.,^202^	Observational case–control study *n* = 13 USA	MCI^a^: 5 HC: 8	VEP latency	MCI: 78 (10) HC: 65.6 (8)	MCI: 80% HC: 50%	Biopac (Biopac Systems Inc., Holliston, MA, USA) ERS100C EEG	NA	MCI: P2 latency↑ vs. HC
Arruda et al.,^203^	Observational case–control study *n* = 20 USA	AD^b^: 45 HC: 60	VEP latency	AD: 73.2 (9) HC: 70.7 (8)	AD: 36% HC: 33%	Biologic Brain Atlas III system	NA	AD: P2 latency↑ vs. HC
Grayson et al.,^204^	Observational case–control study *n* = 20 USA	AD^b^: 10 HC: 10	VEP latency and amplitude	AD: 52‐74 HC: AD matched	–	16‐channel Biologic Model BAllI Brain Atlas Brain Mapping System	NA	AD: latency∼
Haupt et al.,^205^	Observational case–control study *n* = 30 Germany	AD^b^: 15 HC: 15	VEP latency and amplitude	AD: 64.7 (7) HC: 63.4 (6)	AD: 53% HC: 40% (6)	–	NA	AD: latency and amplitude∼
Kromer et al.,^206^	Observational case–control study *n* = 44 Germany	AD: 22 HC: 22	VEP latency	AD: 75.9 (6) HC: 64 (8)	AD: 64% HC: 68%	ROLAND CONSULT RETIport/scan 21 (ROLAND CONSULT, Brandenburg an der Havel, Deutschland)	MMSE score was not significantly correlated with the VEP latencies	AD: latency∼

Abbreviations: AD, Alzheimer's disease; ffERG, full‑field electroretinogram; HC, healthy control; mfERG, multifocal electroretinography; MCI, mild cognitive impairment; MMSE, Mini‐Mental State Examination tests; PERG, pattern electroretinogram; PhNR, Photopic negative response; VEP, visual evoked potential.

^a^ According to the National Institute of Aging and Alzheimer's Association criteria.^164^ ^.^

^b^ Patients were diagnosed with probable Alzheimer's Dementia using the NINCDS‐ADRDA criteria.^165^ ^.^

^c^ Preclinical AD: cognitively healthy with pathological Aβ42/Tau ratio.

^d^ Montreal Cognitive Assessment (MoCA ) < 26 points.

^e^ MCI: Petersen criteria.^166^ ^.^

^f^ Moderate dementia (GDS stage 5); very mild or mild dementia (GDS stages 3 and 4) according to the Global Deterioration Scale (GDS).^207^ ^.^

^g^ The criteria of the Japanese Alzheimer's Disease Neuroimaging Initiative.^208^, ^209^ ^.^

#### 2.4.1 Dynamic vessel analysis (DVA)

DVA measures retinal vascular reactivity by assessing vessel diameter changes to flickering light.^42^ Using a high‐definition camera, the DVA software quantifies both baseline vessel diameters and dynamic responses during alternating cycles of steady and flickering light. Key parameters include maximum dilation and constriction (expressed as a percentage relative to baseline), response times, and areas under flicker‐response curves.^188^ These parameters may indirectly reflect endothelial function of the retinal microvasculature.^40^, ^41^, ^210^ An emerging body of evidence suggests that retinal vascular reactivity can serve as a surrogate biomarker for cerebral microvascular health (Table 3). For instance, a sub‐study from the Maastricht cohort found that retinal arterial dilation in response to flicker light stimulation correlates with pseudo‐diffusion parameters from intravoxel incoherent motion (IVIM) MRI, which reflect microvascular blood flow velocity and tissue architecture in normal appearing white and cortical gray matter.^211^

Given the retinal‐cerebral vascular correlations, recent studies exploring DVA in AD and MCI have yielded conflicting results. Kotliar et al. reported enhanced vascular dilation and delayed arterial responses in AD compared to MCI and controls.^188^ Diagnostic evaluation indicated arterial latency parameters (e.g., time to reach 30% maximal dilation: AUC 0.85, 95% CI: 0.76–0.95) best distinguished AD from controls, while arterial and venous dilation parameters showed moderate discrimination (AUC = 0.77–0.79).^188^ Furthermore, Querques et al. observed reduced arterial dilation and diminished reaction amplitude in AD and MCI, which negatively correlated with CSF amyloid‐β levels (*R* = 0.441–0.580, *p* < 0.05).^189^ However, some studies found no significant group differences in DVA parameters.^190^, ^196^ These discrepancies likely reflect variability in disease classification as well as methodological differences, including inconsistencies in flicker protocols, analysis parameters, and cohort characteristics.

Population‐based studies further support the relevance of retinal microvascular function to cognitive health. In the Maastricht Study (*n* = 3000), a composite microvascular dysfunction score, including DVA parameters, was correlated with global cognitive decline (β = −0.087, *p* < 0.001), equivalent to two additional years of cognitive aging per standard deviation increase in dysfunction.^212^ A pilot study also linked retinal arterial and venous dilation to reaction time and visual processing accuracy, suggesting that retinal vessel responses may reflect domain‐specific cognitive changes.^213^

Current research on DVA and cognitive impairment primarily focuses on its relationships with cognitive function and neuroimaging biomarkers, suggesting DVA‐assessed vascular reactivity holds diagnostic value for detecting cognitive disorders. However, studies investigating clinical subtypes remain preliminary, and standardized assessment parameters are currently lacking. Future studies should establish standardized protocols across distinct clinical stages (e.g., preclinical, MCI, dementia) to optimize DVA utility. Concurrently, rigorous evaluation of clinical feasibility parameters, including diagnostic accuracy, cost‐effectiveness, and added value relative to established biomarkers, is essential to guide implementation in cognitive impairment screening and subtyping.

#### 2.4.2 Electroretinography (ERG)

ERG‐derived parameters, such as the photopic negative response (PhNR) and pattern electroretinogram (PERG), are sensitive to RGC dysfunction, an early event that may precede overt cognitive symptoms^192^ (Table 3).

In a pivotal study by Asanad et al., preclinical AD (as identified by CSF Aβ42/Tau ratios) showed significantly reduced PhNR amplitudes and delayed photopic full‐field ERG a‐/b‐wave implicit times compared to controls achieved 87% sensitivity and 82% specificity (AUC = 0.84, 95% CI: 0.72–0.95) in differentiating preclinical AD.^191^ Earlier research by Katz et al. identified diminished PERG amplitudes in symptomatic AD patients, correlating these changes to optic nerve axonal loss and RGC degeneration.^192^ These findings have been corroborated by subsequent studies suggesting abnormal PERG responses, including reduced wave amplitudes, along with delayed implicit times, in AD patients, with wave amplitude analysis showing moderate to high diagnostic accuracy (AUC = 0.859–0.921).^20^, ^193^, ^195^ Furthermore, ERG smaller amplitudes have been associated with cognitive impairment, assessed via the MoCA, suggesting its potential in evaluating cognition.^194^

It is evident that current research on ERG parameters in cognitive impairment remains limited. However, alterations in wave amplitude show relatively consistent reductions and suggest diagnostic utility for distinguishing AD from HC, with reported AUC values > 0.8. Nevertheless, insufficient sample sizes and study numbers preclude definitive conclusions regarding parameter specificity. Given the technical complexity of ERG relative to structural ophthalmic assessments, whether this approach is better suited for mechanistic investigations of eye–brain connectivity warrants further exploration. Further research should prioritize validating ERG's predictive value for cognitive decline.

#### 2.4.3 Retinal oximetry

Retinal oximetry measures hemoglobin oxygen saturation in retinal blood vessels and offers valuable insights into ocular oxygen metabolism in the context of cognitive impairment (Table 3).

Early studies revealed structural and physiological retinal abnormalities in AD patients. For example, Berisha et al. reported narrowed venous diameter and reduced venous blood flow in AD, which may compromise retinal oxygen delivery.^21^ In the first central nervous system‐focused retinal oximetry study, Einarsdóttir et al. compared 18 patients with mild‐to‐moderate AD with HC, finding significantly higher oxygen saturation in both arterioles (94.2% vs. 90.5%, *p* = 0.028) and venules (51.9% vs. 49.7%, *p* = 0.02) in the AD group.^198^ A follow‐up study by Olafsdottir et al. extended these findings to individuals with MCI, showing increased arteriolar (93.1% vs. 91.1%, *p* = 0.01) and venular (59.6% vs. 54.9%, *p* = 0.001) saturation alongside a reduced arteriovenous difference (33.5% vs. 36.2%, *p* = 0.01), suggesting diminished oxygen extraction by retinal tissue even before clinical dementia onset.^197^ This pattern of a lower arteriovenous difference in oxygen saturation has been consistently observed in both AD and MCI compared to controls. Collectively, these findings support the potential of retinal oxygen saturation parameters and arteriovenous differences associated with cognitive decline.^214^

Unlike conventional methods such as near‐infrared transcranial spectroscopy, which provides only a combined arterial‐venous oxygenation value, retinal oximetry offers separate measurements of arterial and venous saturation, enhancing its clinical applicability.^215^ While altered retinal oxygen patterns are often reported in AD and MCI studies, the evidence has limitations. Most research uses cross‐sectional designs, lacking long‐term data to track changes or predict decline. Studies also have small sample sizes and vary in measurement methods. Future work needs larger, long‐term studies with diverse groups and consistent methods to better understand these oxygen changes in cognitive decline.

#### 2.4.4 Visual evoked potentials (VEPs)

VEPs are electrophysiological measurements that assess the functional integrity of the visual pathways and visual cortex, which are often affected in AD and MCI (Table 3). Multiple studies have reported alterations in VEP components, such as reduced P1 and N1 amplitudes, and prolonged flash VEP‐P2 latency in MCI, particularly during higher‐level visual processing tasks,^199^, ^203^ which are linked to vision‐related cortical dysfunction and correlate with cognitive decline measured by the Mini‐Mental State Examination.^199^ Moreover, MCI showed prolonged higher‐level ventral and dorsal VEP latencies, with dorsal stream stimuli presenting the highest diagnostic accuracy (AUC = 0.831–0.878).^200^ In more advanced stages of AD, VEPs showed prolonged latencies and attenuated amplitudes in components such as P1, P2, N1, and P3, especially in response to emotionally charged or complex visual stimuli,^199^, ^201^, ^202^ while others showed weaker or inconsistent correlations between VEPs changes and cognitive changes.^204^, ^205^, ^206^

Different VEP patterns and stimuli reveal varying associations with cognitive dysfunction. Pattern‐reversal VEPs tend to exhibit reduced amplitudes and prolonged latencies in advanced AD, likely due to retinal neurodegeneration and impaired central processing. Flash VEPs, which engage broader cortical networks, also show latency delays indicative of cortical dysfunction.^22^, ^216^ Additionally, emotional oddball paradigms using VEPs have identified attentional deficits in AD, marked by delayed N2/P3 latencies and hypoactivation in frontal‐occipital networks.^201^ However, the diagnostic specificity of VEPs remains limited by methodological heterogeneity, including variations in stimulus protocols (e.g., chromatic vs. achromatic gratings), recording parameters, and inconsistent control for age‐related visual changes.^200^, ^217^

Future studies should aim to standardize VEP protocols and better clarify their capacity to track functional brain changes across disease stages. This may be achieved through multimodal validation with functional neuroimaging techniques such as functional MRI (fMRI).

#### 2.4.5 Eye movement

Eye movement abnormalities are observed across the stages of cognitive impairment and are closely linked to deficits in specific cognitive domains and disease progression. Among these, saccadic eye movements, particularly prosaccades and antisaccades, serve as key indicators (Table 3).

In individuals with SCD, eye‐tracking tasks**—**including antisaccade, gap saccade, and median fixation paradigms**—**reveal distinct alterations associated with self‐reported cognitive concerns. These abnormalities likely reflect early attentional and executive dysfunction.^218^ Patients with MCI exhibit intermediate eye movement abnormalities that often bridge the spectrum between normal aging and AD. Although antisaccade performance in MCI is generally closer to that of cognitively normal older adults, subtle impairments are evident–such as reduced error correction rates**—**which correlate with frontoparietal cortical thinning.^219^, ^220^, ^221^, ^222^, ^223^, ^224^ These findings suggest early involvement of oculomotor control regions. Prosaccade latency in MCI is typically prolonged relative to HC, and abnormalities in saccadic gain may be more prominent in amnestic MCI subtypes.^221^, ^225^, ^226^

Patients with AD commonly exhibit increased prosaccade latency, reduced velocity, hypometric or misdirected saccades, and greater variability in saccade accuracy and speed. These alterations have been associated with atrophy in the parietal, occipital, and temporal lobes.^219^, ^224^, ^226^, ^227^, ^228^ Antisaccade tasks reveal even more pronounced impairments in AD, including increased error rates, delayed corrective saccades, prolonged latencies, and reduced error correction rates. These findings reflect dysfunction in the frontal lobes and deficits in executive control.^219^, ^224^, ^229^, ^230^, ^231^ Notably, antisaccadic impairments have also been detected in individuals with a first‐degree family history of AD.^232^ Beyond saccadic movements, AD is associated with smooth pursuit dysfunction–characterized by reduced gain and increased saccadic intrusions.^45^, ^227^, ^229^ Abnormalities in fixation have also been reported, including an increased frequency of square wave jerks, greater fixation offsets, and prolonged fixation durations.^44^, ^222^, ^228^, ^233^ In some cases, deficits in the pupillary light reflex, linked to cholinergic dysfunction, have been observed,^234^, ^235^ although such pupillary abnormalities may not be detectable during preclinical stages.^236^

In VCI, particularly those related to CSVD, eye movement disturbances include reduced antisaccade accuracy, prolonged prosaccade reaction times, and abnormal fixation patterns. These deficits are linked to impairments in attention and executive function arising from disruptions in subcortical and frontoparietal networks.^237^, ^238^ Collectively, these oculomotor changes reflect the progressive nature of neural atrophy, synaptic dysfunction, and neurotransmitter deficits**—**particularly involving the cholinergic system**—**across the continuum of cognitive decline.^220^, ^239^

Eye movement parameters suggest variable diagnostic accuracy for distinguishing specific cognitive impairment stages (Figure 1). In SCD, eye‐tracking tasks exhibit high discriminative performance: antisaccade (AUC = 0.911), gap saccade (AUC = 0.904), and median fixation (AUC = 0.891), with a combined gait‐eye‐tracking model achieving an AUC of 0.969.^218^ For MCI, a combination of antisaccade error correction rate and lateral fixation total offset (> 4°) shows good discrimination (AUC = 0.837),^222^ while saccadic gain in antisaccade gap (AUC = 0.70) and overlap tasks (AUC = 0.73) contributes to diagnosis.^225^ Incorporating demographic information, MMSE scores, and eye movement parameters further improves classification accuracy (AUC = 0.840),^221^ while eye‐tracking data alone achieves comparable performance (AUC = 0.845).^240^ In AD, antisaccade accuracy (AUC = 0.800) and memory‐guided saccade accuracy (AUC = 0.798) effectively distinguish AD from HC.^219^ Machine learning (ML) models using smooth pursuit data achieve classification accuracies approaching 95%,^44^ while deep learning combined models achieve an AUC of 0.90.^241^ For CSVD‐related VCI, combining antisaccade accuracy with gait parameters (e.g., stride/swing velocity) yields an adjusted AUC of 0.787.^237^ Variability in discriminative power within diseases may reflect cohort differences in disease severity (e.g., mild vs. moderate stages),^43^ oculomotor paradigm variations,^242^ and analytical heterogeneity‐–ranging from univariate analyses to advanced ML.^23^, ^44^

Wearable technologies, including virtual reality (VR)/augmented reality (AR) headsets and eye‐tracking glasses, hold significant promise for advancing large‐scale adoption of eye movement research and clinical applications. These devices offer several key advantages that support broader implementation. Their portability enables deployment across diverse settings‐–from clinical environments to community‐based contexts**—**facilitating widespread and scalable eye movement data collection.^243^ Head‐mounted displays, integral components of VR systems, are increasingly equipped with high‐precision, low‐latency eye trackers, making them accessible and effective tools for capturing detailed oculomotor parameters.^244^ Additionally, the ability to create controlled, immersive virtual environments minimizes environmental variability, ensuring consistent and comparable eye movement assessments across individuals and settings.^245^ Operational convenience, including short, self‐administered tasks, further enhances participant compliance and study feasibility.^243^ Eye‐tracking glasses, meanwhile, enable the capture of naturalistic eye movements in real‐world scenarios, thus broadening the scope and ecological validity of data collection.^246^, ^247^ Despite these advantages, several limitations hinder widespread adoption. Hardware incompatibility with clinical disinfection protocols restricts use in healthcare settings, and persistent challenges related to eye‐tracking data quality and calibration**—**particularly among older adults with AD**—**compromise data reliability and utility.^244^, ^246^

In general, eye movement research in the field of cognitive impairment has been explored relatively more extensively compared to other ocular parameters. Alterations in antisaccade, prosaccade, and fixation paradigms emerge during the SCD stage and persist through AD, suggesting diagnostic utility across disease stages. Combined oculomotor parameters or their integration with clinical indicators show improved discriminative capacity (AUC > 0.8), though this approach currently lacks robust verification. In VCI, antisaccade and fixation changes also occur, suggesting that the specificity of oculomotor parameters for distinguishing AD from VaD requires further investigation. There is also a paucity of studies on the correlation between eye movement parameters and changes in brain function across different stages, and a research gap remains regarding asymptomatic populations with positive biomarkers. Meanwhile, current studies are limited by small sample sizes and a lack of longitudinal research. The integration of eye movement indicators with emerging technologies may facilitate more convenient and comprehensive assessments. Future research should involve larger prospective cohorts, refine and standardize the detection of different indicators, and compare the detection capabilities and clinical benefits of various emerging technologies with traditional desktop eye trackers. This will help promote their clinical application.

### 2.5 Molecular biomarkers in the retina

**FIGURE 2:**
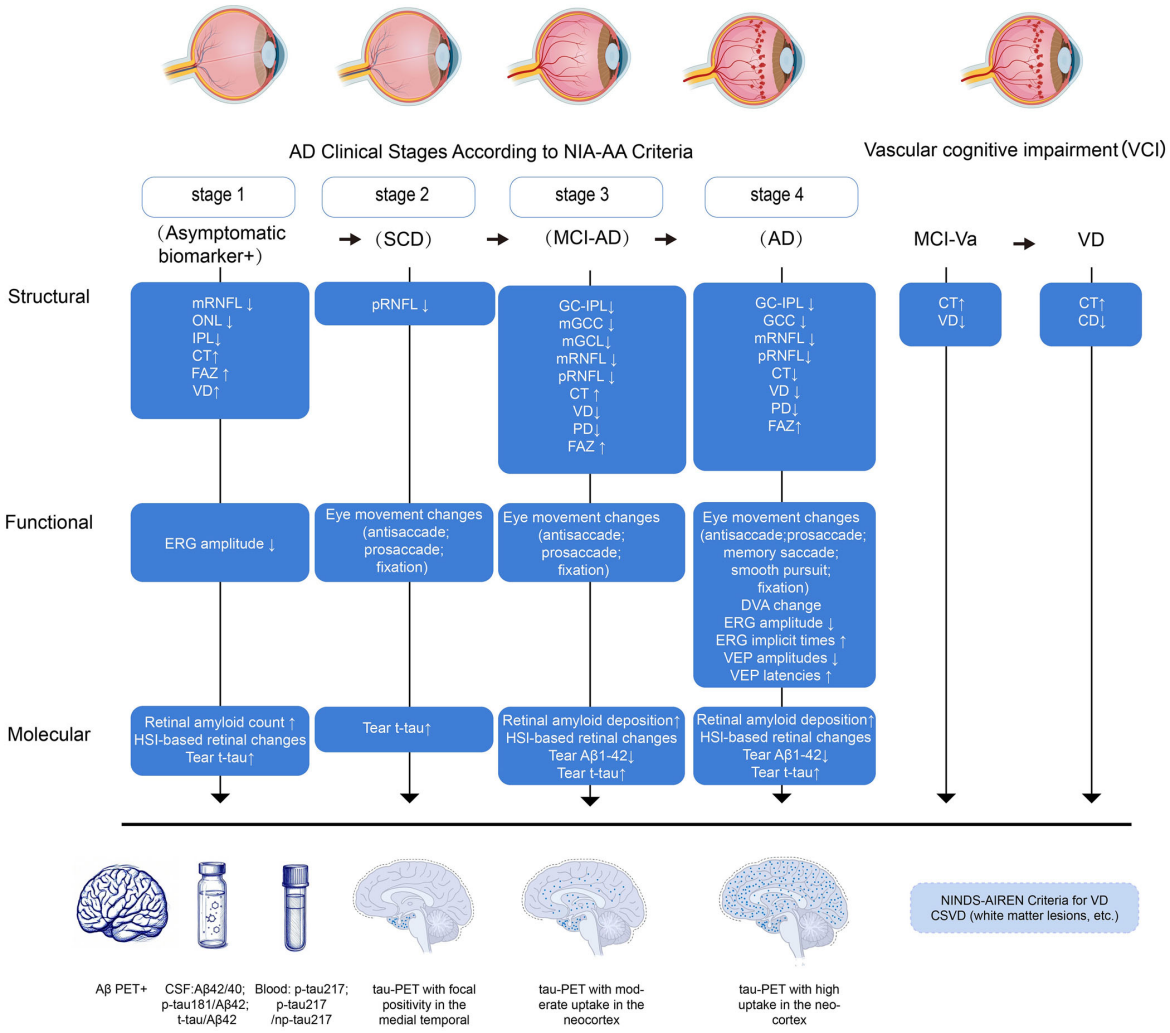
Retinal biomarkers and brain changes during different clinical stages and types of cognitive impairment.

Retinal amyloid‐beta (Aβ) detection shows diagnostic potential for early AD, with deposits in ganglion cells paralleling cerebral pathology and correlating with visual disturbances.^248^ In transgenic AD mouse models, retinal Aβ plaques emerge as early as 2.5 months, preceding cerebral Aβ accumulation,^19^ suggesting that the retina may reflect preclinical AD. Recent advances have suggested that various ocular detection Aβ methods, including curcumin‐based retinal Aβ imaging, hyperspectral imaging (HSI), and Aβ measurement in the lens, aqueous humor (AH), and tears, have been shown to correlate with cognitive decline (Table 4). These findings suggest ocular Aβ burden may reflect associated brain pathology (Figure 2).

**TABLE 4:** Key studies on the association between retinal molecular biomarkers and cognitive function.

Author	Cohort information	Sample	Retinal parameter	Age	Gender character (Female, %)	Retinal imaging	Cognitive outcome	Summary of results
Curcumin‐based retinal Aβ imaging								
Tadokoro et al.,^249^	Observational case–control study *n* = 30 Japan	AD^b^: 13 MCI^b^: 7 HC: 10	Quantitative analysis of retinal amyloid spots	AD: 77.6 (4) MCI: 73.9 (7) HC: 75.7 (4)	AD: 61.5% MCI: 42.9% HC: 40%	Autofluorescence mode of a scanning laser ophthalmoscope (SLO; SPECTRA LIS, Heidelberg Engineering, Heidelberg, Germany)	NA	AD: Retinal amyloid deposition↑vs. HC MCI: Retinal amyloid deposition↑vs. HC
Ngolab et al.,^250^	Observational case–control study *n* = 8 USA	Aβ (‐): 4 Aβ (+): 4	Retinal amyloid spots	Aβ (‐): 69 Aβ (+): 69	Aβ (‐): 50% Aβ (+): 50%	The RETIA 2 imaging system (EyeCare)	Retinal amyloid deposition was not correlated with the cognitive score of the MMSE	Aβ (+): retinal amyloid spots↑
den Haan et al.,^251^	Observational case–control study *n* = 40 Netherland	AD^a^: 26 HC: 14	Retinal amyloid spots	AD: 67 (9) HC: 71 (12)	AD: 38% HC: 71%	Heidelberg Engineering Spectralis Spectral Domain Scanning Laser Ophthalmoscope	NA	Visual assessment showed no difference between AD patients and controls for pre‐ and post‐curcumin images
Dumitrascu et al.,^252^	Observational case–control study *n* = 28 USA	CI (MoCA≤26): 19 HC: 9	Amyloid plaque distribution	CI: 65 (8) HC: 65 (5)	CI: 47% HC: 56%	Confocal scanning laser ophthalmoscope (SLO Retia, CenterVue SpA)	Peri‐venular AP count correlated with clinical dementia rating and MocA	CI: peri‐venular amyloid plaque count↑
Dumitrascu et al.,^253^	Observational case–control study *n* = 34 USA	CI (MoCA≤26): 16 HC: 18	Retinal amyloid count	CI: 66.9 (7) HC: 63.5 (7)	CI: 38% HC: 67%	Confocal scanning ophthalmoscope (Retia, CenterVue SpA)	NA	CI: The proximal mid‐periphery retinal amyloid count and retinal amyloid areas ↑
Hyperspectral imaging (HSI) of retinal amyloid‐β								
Sharafi et al.,^254^	Observational case–control study *n* = 46 Canada	CI^c^: 20 (13 with Aβ+) HC: 26 (3 with Aβ+)	Texture measures	All: 60–85	–	MHRC	NA	Aβ+ differences in texture measures were observed in the spectral range 450–550 nm
More et al.,^255^	Observational case–control study *n* = 35 USA	CI: 19 HC: 16	rHSI signature	CI: 77.3 (7) HC: 68.2 (6)	CI: 11% HC: 81%	Topcon TRC‐50 EX mydriatic camera	NA	The largest spectral deviation from control subjects, rHSI signature, was obtained at the MCI stage with MMSE scores ⩾22
Thach et al.,^256^	Observational case–control study *n* = 35 Netherland	PET‐Aβ+: 25 (preclinical AD: 21; AD: 4) PET‐Aβ‐: 6	Spatial‐spectral features	PET‐Aβ+: 72.8 (5) PET‐Aβ‐: 67.8 (9)	PET‐Aβ+: 52% PET‐Aβ‐: 33%	MHRC	NA	Three spatial‐spectral features were identified for the classification of the cerebral amyloid‐PET status
Poudel et al.,^257^	Observational case–control study *n* = 66 Netherland	PET‐Aβ+: 32 PET‐Aβ‐: 34	Retinal Spectral Reflectance within 450–585 nm	PET‐Aβ+: 71.7 (6) PET‐Aβ‐: 67.4 (6)	PET‐Aβ+: 6% PET‐Aβ‐: 9%	MHRC	NA	The retinal features in the superior view showed higher inter‐subject variability in Aβ+
Lemmens et al.,^258^	Observational case–control study *n* = 39 Belgium	AD^a^: 17 HC: 22	Performance of the selected ROIs	AD: 71.9 (7) HC: 68.6 (8)	AD: 59% HC: 41%	XIMEA SNm4x4 VIS hyperspectral snapshot camera	NA	Values of two regions of interest were different between AD and controls
Hadoux et al.,^259^	Observational case–control study *n* = 35 Canada	PET‐Aβ+: 15 PET‐Aβ‐: 20	Retinal reflectance spectra	PET‐Aβ+: 68.5 (8) PET‐Aβ‐: 69.1 (2)	PET‐Aβ+: 87% PET‐Aβ‐: 65%	MHRC	NA	Significant differences in the retinal reflectance spectra were found between individuals with PET‐Aβ+ and controls

Abbreviations: Aβ, amyloid‐beta; AD, Alzheimer's disease; CI, cognitive Impairment; HC, healthy controls; MCI, mild cognitive impairment; MHRC, metabolic hyperspectral retinal camera; MMSE, Mini‐Mental State Examination tests; PET, positron emission tomography; PVEP, pattern visual evoked potential.

^a^ According to the National Institute of Aging and Alzheimer's Association criteria.^164^ ^.^

^b^ The criteria of the Japanese Alzheimer's Disease Neuroimaging Initiative.^208^, ^209^ ^.^

^c^ CI: Values outside the normal range for the Mini–Mental State Examination and/or the Montreal Cognitive Assessment.^254^ ^.^

#### 2.5.1 Curcumin‐based retinal Aβ imaging

Curcumin, a naturally fluorescent polyphenol derived from turmeric, serves as a potential probe for imaging retinal Aβ plaques due to its binding affinity for Aβ aggregates and its ability to cross the blood–brain and blood–retinal barriers.^260^ It binds to Aβ aggregates by interacting with specific fibril grooves and disrupting β‐sheet formation in Aβ monomers. Curcumin's propensity for self‐aggregation, when associated with Aβ binding, may contribute to localized fluorescence at deposition sites, which has been visualized using scanning laser ophthalmoscopy (SLO) equipped with tailored excitation and emission filters.^19^, ^261^, ^262^

Quantification of retinal Aβ load, typically reported as retinal amyloid count (RAC) or total amyloid area, has been reported in some studies to correlate with cognitive decline and neuropathological features. In preclinical AD, higher RAC levels have been found to correlate with increased cerebral Aβ burden measured by PET standardized uptake values.^250^ Furthermore, retinal amyloid deposition was greater in AD and MCI than in HC, and this deposition correlated with whole‐brain gray matter atrophy but not with medial temporal lobe atrophy, implying that retinal Aβ may reflect visual cortex pathology.^249^ Notably, deposits in superotemporal quadrant peri‐vascular region have been linked to reduced hippocampal volume, lower cognitive scores, and increased white matter hyperintensities.^248^, ^252^, ^253^ Peri‐arteriolar Aβ is generally more abundant than peri‐venular Aβ across cognitive states, while the presence of secondary venular Aβ has shown specific associations with dementia severity.^252^ However, findings across studies exhibit inconsistencies. As an example, den Haan et al. reported no significant differences in retinal fluorescence between AD patients and controls using three different oral curcumin formulations.^251^

The commercial viability of curcumin‐based retinal imaging tools is hindered by critical formulation issues inherent to oral curcumin. Native curcumin exhibits extremely low oral bioavailability (< 1%) due to poor aqueous solubility, rapid hepatic metabolism, and intestinal glucuronidation, necessitating specialized formulations for reliable signal detection.^263^, ^264^ To address this, three generations of bioavailability‐enhanced formulations have emerged: first‐generation (e.g., curcumin‐piperine combinations inhibiting metabolism), second‐generation (emulsifier‐based systems like Theracurmin and Novasol), and third‐generation (natural complex‐based systems like Longvida enabling “free” curcumin delivery).^265^, ^266^, ^267^ Nanoparticle‐encapsulated (e.g., NanoCurc) and liposomal formulations further enhance stability and tissue penetration,^268^, ^269^ though comparative efficacy data across formulations remains limited.^270^

Current research on retinal Aβ deposition and curcumin interventions in cognitive impairment faces substantive limitations, including an unresolved temporal sequence linking retinal Aβ accumulation to cognitive decline, inadequate comparative studies of curcumin formulations, and persistent methodological inconsistencies across investigations. Future research should prioritize larger‐scale studies and optimizing curcumin formulations for consistent uptake and signal strength to facilitate clinical translation, verify whether fundus changes parallel pathological alterations in the brain, and further explore the utility of retinal amyloidosis as a predictive tool for cognitive decline.

#### 2.5.2 Hyperspectral imaging (HSI)

Hyperspectral imaging reveals parameters associated with retinal Aβ pathology by analyzing spectral shifts in light scattering. This technique involves illuminating the retina with visible to near‐infrared light and capturing the reflected light to generate spatial‐spectral data cubes. Aβ oligomers alter Rayleigh scattering, producing decreased reflectance in the 480–550 nm range, a spectral signature attributed to Aβ accumulation. Unlike traditional imaging methods, HSI leverages Aβ’s unique light‐scattering properties without requiring extrinsic fluorescent probes.^47^, ^259^, ^271^ ML algorithms help extract Aβ‐specific spectral features, enabling the detection of cerebral amyloid status.^254^, ^259^, ^262^

HSI‐derived retinal changes associated with Aβ and phosphorylated tau (pTau) show distinct patterns across disease stages.^255^, ^272^ In preclinical stages prior to cognitive decline, APP/PS1 mice exhibit progressive short‐wavelength reflectance reductions from 3 to 8 months, while cognitively unimpaired humans with elevated cerebral Aβ show analogous 450–550 nm spectral changes.^255^, ^273^ Distinct spectral profiles have also been observed in cognitively impaired individuals, with the most pronounced differences seen in early‐stage MCI.^255^ In clinical stages, HSI‐based classifiers suggest robust performance: AUC values range from 0.745 to 0.891 for distinguishing varying amyloid levels.^257^ Additionally, spatial‐spectral features outperform morphological features, with a classifier integrating these features achieving 85% accuracy in determining amyloid‐PET positivity.^254^, ^256^ HSI yields an AUC of 0.82 for differentiating MCI from HC.^259^ When combined with RNFL thickness, this approach achieves an AUC of 0.74 (95% CI: 0.60–0.89) for distinguishing AD from HC.^258^ Additionally, HSI scores are associated with cerebral Aβ burden on PET and with gray matter atrophy.^259^, ^262^

HSI parameters correspond to cerebral alterations from the preclinical stage and may indicate modest diagnostic utility in both preclinical stage and MCI (Figure 2). Despite its potential, the evidence base is largely cross‐sectional, lacking sufficient longitudinal data to validate the use of HSI in tracking disease progression. Further research should evaluate the feasibility of implementing HSI for retinal pathology screening in population‐based settings, alongside validation of its diagnostic accuracy. Spectral changes within the 450–550 nm range appear most common and potentially closely correlated with cerebral Aβ, but require further validation to establish consistent biomarkers. Additionally, the relative suitability of more practical systems like snapshot HSI for widespread implementation needs to be assessed.

#### 2.5.3 Detection of Aβ in the lens, aqueous humor, and tears

The crystalline lens, a transparent structure critical for light focusing, has been investigated as a potential site for Aβ biomarker detection in AD. Studies have identified Aβ1‐40 and Aβ1‐42 deposits in human lenses, particularly in AD patients, where they form electron‐dense aggregates with supranuclear and deep cortical lens fiber cells, at concentrations comparable to those in the brain.^274^ Animal models, including monkeys, rats, and transgenic mice, also show lens Aβ deposition.^275^, ^276^ Non‐invasive imaging techniques, such as fluorescent ligand eye scanning (FLES), have shown high sensitivity (85%) and specificity (95%) in differentiating AD patients from controls, with lens Aβ levels correlating with cerebral amyloid burden via PET signals^48^, ^277^ (Figure 2). However, not all findings are consistent. Some *post mortem* studies failed to detect lens Aβ in AD patients using immunohistochemistry, possibly due to methodological differences in tissue processing, staining protocols, or Aβ epitope accessibility.^278^, ^279^ Despite ongoing debate regarding Aβ’s presence in the lens, its strong influence by age and ocular comorbidities necessitates further clinical validation.

The AH, a clear fluid vital for ocular function,^280^ is another emerging source for AD biomarkers. Proteomic studies have detected Aβ40 and Aβ42 in AH, likely originating from the retina or vitreous.^281^ The structural similarities between the blood–AH and blood–CSF barriers^282^ support the idea that AH may reflect CNS pathology. Experimental models show that Aβ introduced into CSF can rapidly migrate to AH, and unlike CSF or blood, AH Aβ levels remain stable with age.^49^ These findings indicate that the aqueous humor could represent a minimally invasive compartment for brain Aβ pathology. Clinical validation further supports its relevance. One study quantified AD biomarkers in human AH, suggesting that elevated neurofilament light chain (NfL) and phosphorylated tau (p‐tau181) were associated with cognitive decline (lower MMSE scores).^283^ Combined with retinal imaging, AH biomarker analysis represents a compelling strategy for early, non‐invasive AD diagnosis and progression monitoring.

Tear fluid, an easily accessible biofluid, has also shown promise for detecting Aβ and tau in AD and cognitive impairment. Tear t‐tau levels were significantly elevated in AD compared to those with SCD and may reflect progressive elevation correlating with advancing clinical pathology severity^284^ (Figure 2). However, tear Aβ1‐42 levels were significantly reduced in AD and MCI patients compared to HC.^285^ This biomarker showed strong diagnostic performance in distinguishing MCI and AD from HC, with 93% specificity and 81% sensitivity (AUC = 0.91).^285^ Furthermore, Aβ1‐42 concentrations showed inverse correlations with psychometric test scores and cortical thinning measures.^285^ Electrochemical biosensors developed for tear‐based Aβ detection offer high sensitivity (1–100 pg/mL), with tear Aβ concentrations reported to be approximately 10‐fold higher than in blood.^286^ The ongoing TearAD study aims to validate tear Aβ biomarkers in a large longitudinal cohort, integrating tear analysis with neuroimaging and cognitive assessments to confirm its diagnostic value, minimal invasiveness, and cost‐effectiveness.^50^ Despite potential, tear‐based AD diagnostics face methodological challenges like inconsistent collection protocols and variable Aβ quantification.^287^ Nevertheless, the detection of AD‐related proteins in tears and their potential correlation with cognitive impairment support opportunities for early, cost‐effective clinical adoption.

## 3 CURRENT STATUS AND LIMITATIONS

In summary, current studies on retinal biomarkers for cognitive impairment have identified multiple indicators, suggesting shared pathological pathways between retinal and cerebral systems in disease progression. The revised 2024 National Institute on Aging‐Alzheimer's Association (NIA‐AA) criteria^288^ emphasize the pivotal role of tau‐PET in biological staging. In individuals with abnormal tau‐PET, clinical symptoms and neurodegeneration show close spatiotemporal and functional correlations with tau‐PET uptake patterns (location/magnitude), but not with amyloid‐PET.^289^ The updated framework also proposes an integrated biological‐clinical staging scheme, reflecting the relationship between AD pathological stage and clinical severity along typical progression trajectories. We integrated the characteristic biological‐clinical staging changes with corresponding fundus biomarker alterations, as summarized below (Figure 2).

In the preclinical stage of AD‐related cognitive impairment, patients lack clinical symptoms but exhibit positive biomarkers (Aβ‐PET+, CSF: Aβ42/40 ratio; p‐tau181/ Aβ42; t‐tau/ Aβ42, Blood: p‐tau217; p‐tau217/np‐tau217).^288^ During this phase, retinal changes may suggest compensatory increases in VD, FAZ, and ERG alterations. Curcumin‐enhanced imaging and HSI suggest Aβ accumulation, while tear analysis reveals elevated t‐tau levels. SCD represents a distinct clinical phase characterized by patient‐reported cognitive concerns, with tau‐PET positivity typically emerges in the medial temporal lobe,^288^ paralleled by increased t‐tau concentrations in tears. Limited data exist on other retinal biomarkers during SCD, though eye movement abnormalities may manifest nonspecifically. During MCI and AD stages, tau‐PET shows progressive neocortical involvement.^288^ Structural retinal parameters (GC‐IPL, GCC, RNFL, VD, PD, FAZ) exhibit comparable alterations in both stages. Eye movement tracking shows diagnostic potential for both stages, while ERG, VEP, and retinal oximetry reveal more functional changes in AD. Tear analyses reveal increased t‐tau concentrations and decreased Aβ1‐42 levels in MCI and AD, correlating with brain pathological progression, though further validation is required to confirm these findings. Additionally, VCI exhibits distinct neuropathological features from ADCI. In our study, retinal biomarkers for VCI diagnosis were evaluated using the NINDS‐AIREN criteria^163^ or cognitive deficits linked to vascular etiologies such as CSVD. CT and CD measurements suggest potential diagnostic utility for VCI detection in this context.

Regarding stage‐discrimination capabilities (Figure 1), structural biomarkers have been most extensively studied. Parameters such as VD, PD, and FAZ show relatively consistent alterations, though their distinguishing ability remains limited. Functional parameters, particularly eye movement tracking, show better stage‐separation performance, but lack standardized protocols across studies. Molecular indicators (e.g., tear T‐tau, HSI‐derived retinal changes) may reflect underlying cerebral pathology, but their clinical validity requires further validation due to limited studies and a lack of longitudinal evidence. New integrated or AI‐enhanced approaches show promising diagnostic potential but warrant additional investigation.

Retinal biomarkers offer potential for integration with cerebral clinical and pathological indicators across cognitive impairment stages. However, current studies face limitations including methodological heterogeneity, substantial confounding variables (e.g., shared retinal–cerebral risk factors), inter‐cohort diversity, and insufficient longitudinal data. Consequently, no uniformly applicable clinical biomarkers have been established.

## 4 FUTURE DIRECTIONS

**FIGURE 3:**
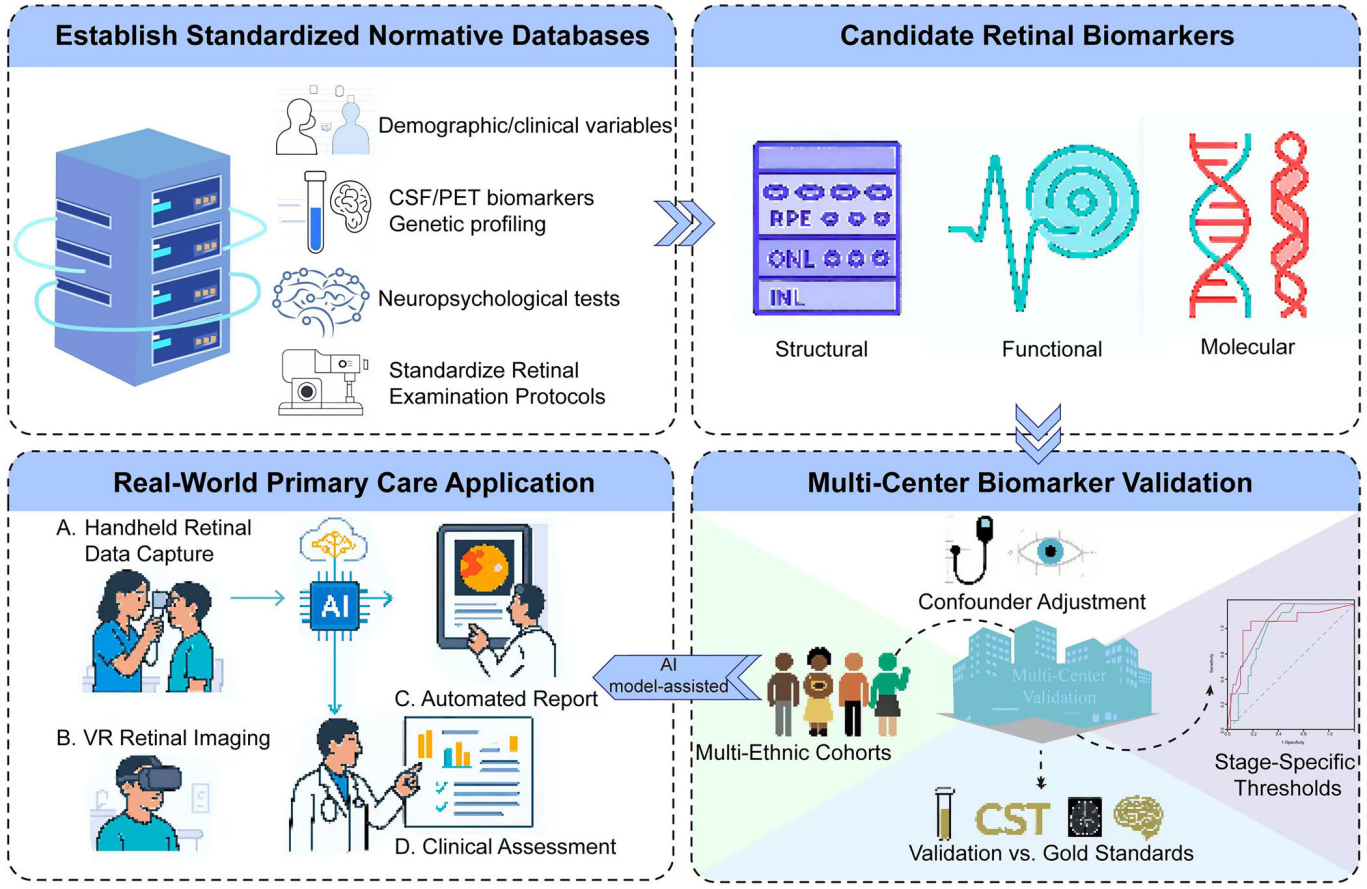
Clinical translation roadmap for retinal biomarkers.

To advance retinal and cognitive impairment research and facilitate clinical translation, future efforts should prioritize four key phases (Figure 3): First, establish standardized databases with detailed clinical profiles, validated cognitive biomarkers, and neuroimaging to define multi‐stage cohorts, while implementing harmonized protocols for multimodal retinal assessments (structural, functional, and molecular); Second, identify candidate retinal biomarkers through comparative analysis of these databases against existing evidence; Third, conduct longitudinal multi‐center studies across diverse populations to validate biomarkers, control for confounders (age, ethnicity, systemic diseases), analyze diagnostic performance against gold‐standard measures, and develop integrated diagnostic indicators through AI‐assisted synthesis of multimodal retinal and clinical data; Finally, optimize clinical implementation with non‐invasive, cost‐ and time‐efficient methods—such as handheld fundus cameras for structural parameters and VR‐integrated eye‐tracking for functional assessments—and establish integrated point‐of‐care platforms to triage patients for advanced diagnostics and clinical decision support.

## 5 CONCLUSION

Evidence suggests associations between retinal parameters and cognitive status, though important gaps persist regarding diagnostic specificity, clinical applicability, and causal mechanisms. Addressing these limitations through rigorous, mechanistically grounded research may advance the application of retinal assessments for detecting early cognitive decline, stratifying risk, and monitoring disease progression.

## CONFLICT OF INTEREST STATEMENT

The authors declare no conflicts of interest. Author disclosures are available in the Supporting Information.

## Supporting information



Supporting Information
